# Transcriptomic and Macroscopic Architectures of Multimodal Covariance Network Reveal Molecular–Structural–Functional Co-alterations

**DOI:** 10.34133/research.0171

**Published:** 2023-06-08

**Authors:** Lin Jiang, Yueheng Peng, Runyang He, Qingqing Yang, Chanlin Yi, Yuqin Li, Bin Zhu, Yajing Si, Tao Zhang, Bharat B. Biswal, Dezhong Yao, Lan Xiong, Fali Li, Peng Xu

**Affiliations:** ^1^ The Clinical Hospital of Chengdu Brain Science Institute, MOE Key Lab for Neuroinformation, University of Electronic Science and Technology of China, Chengdu 611731, China.; ^2^ School of Life Science and Technology, Center for Information in BioMedicine, University of Electronic Science and Technology of China, Chengdu 611731, China.; ^3^ School of Psychology, Xinxiang Medical University, Xinxiang 453003, China.; ^4^ School of Science, Xihua University, Chengdu 610039, China.; ^5^ Department of Biomedical Engineering, New Jersey Institute of Technology, Newark, NJ, USA.; ^6^ School of Electrical Engineering, Zhengzhou University, Zhengzhou 450001, China.; ^7^ Research Unit of NeuroInformation, Chinese Academy of Medical Sciences, 2019RU035, Chengdu, China.; ^8^ Montreal Neurological Institute and Hospital, McGill University, Montreal, QC, Canada.; ^9^ Department of Electrical and Computer Engineering, Faculty of Science and Technology, University of Macau, Macau, China.; ^10^ Radiation Oncology Key Laboratory of Sichuan Province, 610041 Chengdu, China.; ^11^ Rehabilitation Center, Qilu Hospital of Shandong University, Jinan 250012, China.

## Abstract

Human cognition is usually underpinned by intrinsic structure and functional neural co-activation in spatially distributed brain regions. Owing to lacking an effective approach to quantifying the covarying of structure and functional responses, how the structural–functional circuits interact and how genes encode the relationships, to deepen our knowledge of human cognition and disease, are still unclear. Here, we propose a multimodal covariance network (MCN) construction approach to capture interregional covarying of the structural skeleton and transient functional activities for a single individual. We further explored the potential association between brain-wide gene expression patterns and structural–functional covarying in individuals involved in a gambling task and individuals with major depression disorder (MDD), adopting multimodal data from a publicly available human brain transcriptomic atlas and 2 independent cohorts. MCN analysis showed a replicable cortical structural–functional fine map in healthy individuals, and the expression of cognition- and disease phenotype-related genes was found to be spatially correlated with the corresponding MCN differences. Further analysis of cell type-specific signature genes suggests that the excitatory and inhibitory neuron transcriptomic changes could account for most of the observed correlation with task-evoked MCN differences. In contrast, changes in MCN of MDD patients were enriched for biological processes related to synapse function and neuroinflammation in astrocytes, microglia, and neurons, suggesting its promising application in developing targeted therapies for MDD patients. Collectively, these findings confirmed the correlations of MCN-related differences with brain-wide gene expression patterns, which captured genetically validated structural–functional differences at the cellular level in specific cognitive processes and psychiatric patients.

## Introduction

Multimodal fusion takes advantage of complementary information from multiple neurophysiological techniques, e.g., functional magnetic resonance imaging (fMRI), diffusion MRI (dMRI), structural MRI (sMRI), and electroencephalography (EEG). This will help us explore the complex interplay of structural–functional alterations in the brain of healthy controls (HCs) [1] or patient populations [2]. Despite the increased effort in exploring multimodal fusion, unimodal approaches are still the mainstream when investigating the covariance networks (CNs), as CNs usually reflect the interindividual covariation in brain morphology of different brain areas [3].

The integration of functional and structural modalities helps deepen our understanding of cognitive processing, as well as improve clinical diagnostics [4,5]. EEG in particular provides excellent temporal resolution for the continuous reading out of the brain’s electrical activity, which is advantageous to probing the fine-grained temporal process of human cognition [6]. In this regard, our previous study investigating data fusion from multiple modalities has demonstrated that the addition of EEG improved analytical sensitivity [7]. Moreover, there is a consistent degree of topological isomorphism between whole-brain functional and structural networks [8], and the structural–functional couplings are not fixed but rather present significant flexibility during task execution [9] and dynamic reorganization in brain diseases [10]. Hence, rather than constructing CNs by unimodal information, in our current work, individual multimodal CN (MCN) is proposed to capture the interregional structural–functional covarying patterns from multiple modalities. Methodologically, as an extension of the morphometric similarity network [11], MCN can be constructed for a single individual and potentially used to capture the brain’s altered structural–functional covariance related to specific cognitive processes (e.g., decision-making) or psychiatric disorders [e.g., major depression disorder (MDD)].

Manifestation of human brain networks in both functional [12] and structural connectomes [13] is highly heritable, implying that brain connectivity is genetically encoded. In this regard, functional connectivity has been proposed to be encoded by the transcriptome, which could be the underlying mediating factor between the human genome and cognition [14]. In addition, structural organization underlies and constrains functional connections, and the latter may be a more suitable mediator than structural connectivity between genes and cognitive processes and behaviors [14]. Hence, accurately quantifying the interregional covariant relationship between the brain’s transient functional activities and structural framework by MCN is vital to understanding how brain circuits interact to satisfy the needs of cognitive functions, and how genes encode such interactions.

Establishing how neurobiological processes combine to underpin human cognition requires multiscale and multimodal information, including genetic and cell-specific transcriptomic assessments, as well as brain functional and structural datasets. In recent decades, human brain imaging genetics appeared as a promising approach to exploring the molecular mechanism of the structural and functional connectome organizations [15]. Particularly, the Allen Human Brain Atlas (AHBA) has been largely applied to probe brain transcriptomic changes related to various brain connectivity in human neuroimaging [16]. Specifically, multiple lines of evidence have reached a consensus regarding the relationship between conserved gene expression patterns and structurally/functionally relevant circuits underlying brain cognition [17,18]. The combined neuroimaging and gene expression datasets have also provided insight into how pathological alteration in the cellular architecture through transcriptomic dynamics drives macroscale functional and structural brain network changes in different disease conditions [19], such as schizophrenia [20]. To date, the relationship between cognition and disease-specific structural–functional covarying patterns with corresponding molecular and cellular alterations in the human brain remains largely unknown.

Understanding how macroscopic structural–functional connectomes are intertwined to influence cognition and behavior through genes, cells, circuits, and networks is an important and promising research field in human neuroscience. Here, we first develop an approach to construct MCN for a single individual by combining multiple modalities of MRI with electrophysiological signals, aiming to capture the structural–functional covarying patterns for different brain states or disease conditions. Once the replicability and robustness of individual MCNs were assessed in 2 independent datasets, partial least squares (PLS) regression was performed to map these MCN variations to anatomically patterned gene expression from the AHBA. Finally, functional enrichment analyses were implemented to illustrate the aggregated ontological pathways for cognition- or disease-specific genes, which were further linked to specific cell types contributing most to the transcriptomic variations corresponding to the alterations in MCN.

## Results

### Study design and overview

**Fig. 1.**
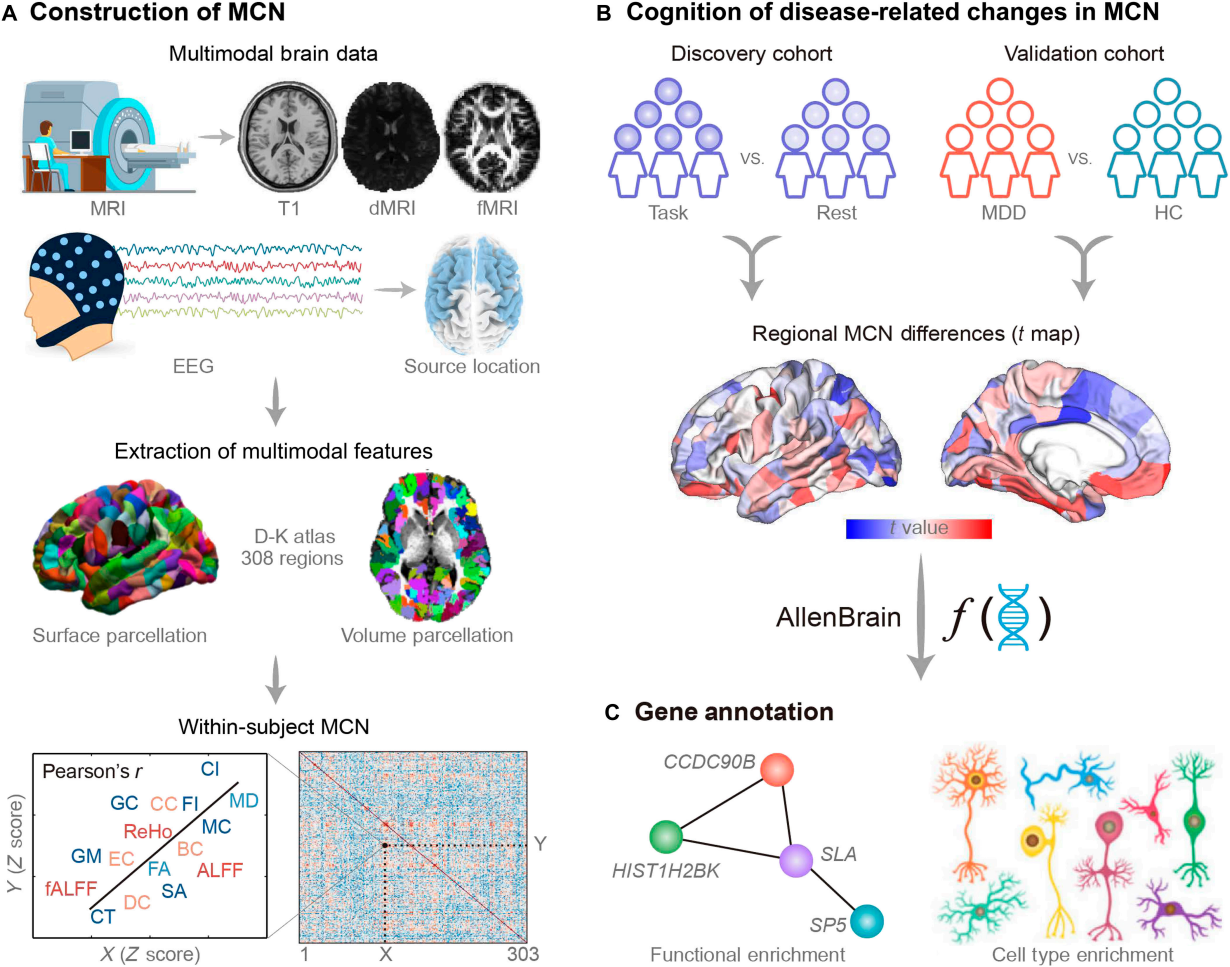
Overview of the analysis pipeline. (A) Construction of multimodal covariance network (MCN) for a single individual. MCN was constructed across multimodal structural and functional information that were parcellated by the Desikan–Killiany (D-K) atlas (a total of 308 regions). (B) Cognition- or disease-related changes in MCN. Two independent cohorts were included: Task-evoked MCN alterations were investigated in the discovery cohort, while the validation cohort probed the MDD-related MCN changes. (C) Gene annotation. Transcriptome and MCN associations were first established by partial least squares (PLS) regression, based on which enrichment analyses were implemented on genes related to the first component of PLS (PLS1).

In this study, neuroimaging, electrophysiological, and transcriptomics data were integrated to investigate the interregional structural–functional covariance by MCN and to determine potential associations between MCN and gene expression (Fig. 1). Two independent cohorts, a discovery cohort recruiting healthy participants to perform a classical gambling task and a resting-state validation cohort of MDD from the EMBARC Project [Establishing Moderators and Biosignatures of Antidepressant Response in Clinical Care, National Institute of Mental Health (NIMH)-funded multi-site clinical trial project for MDD] [21], were included to explore these associations. First, the resting-state multimodal datasets of healthy participants in both cohorts were applied to test the capacity and validate the replicability of our developed MCN in capturing interregional structural–functional covarying in the human brain (Fig. 1A). Then, for the discovery cohort, we established the relationship between task-evoked MCN changes (Fig. 1B) and whole-brain gene expressions adopting the AHBA dataset, and further extracted a list of genes underlying task-related structural–functional covarying. Finally, we explored the relationships underlying MDD-MCN abnormalities in the validation cohort with the AHBA transcriptomic data to probe the molecular mechanism of brain structural–functional changes in MDD patients (Fig. 1B and C).

### Replicable multimodal covariance patterns in 2 cohorts

**Fig. 2.**
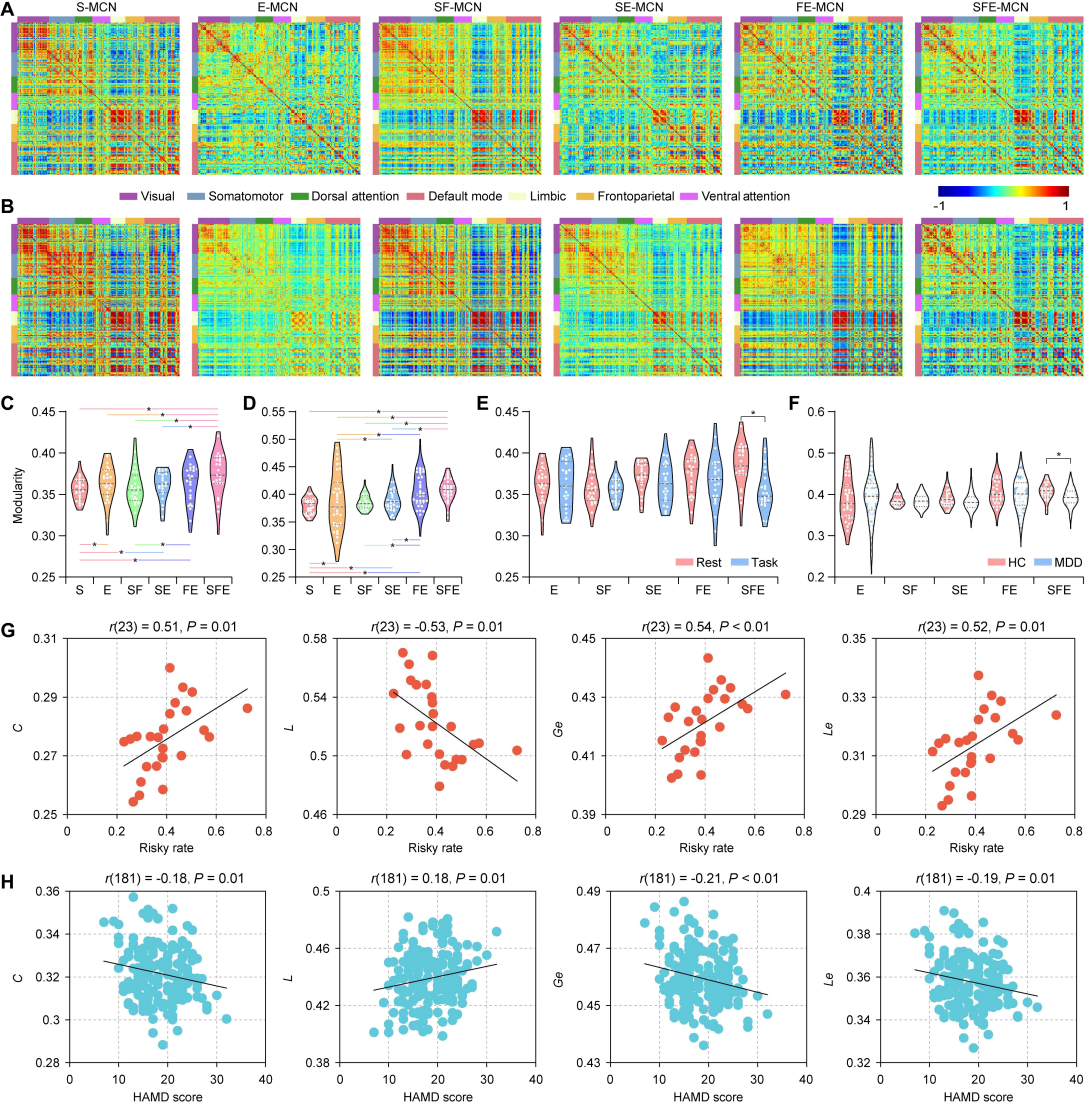
MCN in the discovery and validation cohort. (A) Resting-state MCNs of the discovery cohort. (B) Resting-state MCNs of the validation cohort. S-MCN: MCN constructed by sMRI; E-MCN: MCN constructed by EEG; SF-MCN: MCN constructed by sMRI and fMRI; SE-MCN: MCN constructed by sMRI and EEG; FE-MCN: MCN constructed by fMRI and EEG; SFE-MCN: MCN constructed by sMRI, fMRI, and EEG. (C) Modularity of various MCNs in the discovery cohort. (D) Modularity of various MCNs in the validation cohort. The colored violins represent the modularity of S-MCN, E-MCN, SF-MCN, SE-MCN, FE-MCN, and SFE-MCN, respectively, and the binary color-coded line with asterisk reflects a significant modularity difference between 2 corresponding MCNs represented by the 2 colors (*P* < 0.05). (E) MCN modularity of the resting state and gambling task in the discovery cohort. (F) MCN modularity of HCs and MDDs in the validation cohort. The asterisk indicates the significant difference in the modularity (*P* < 0.05). (G) Relationships between the task SFE-MCN properties and risky rates in the discovery cohort. (H) Relationships between the SFE-MCN properties of the MDD and HAMD scores in the validation cohort. The black line represents the fitted curve, and the colored circles denote the participants.

For both cohorts, we first evaluated the structural MCNs (S-MCNs) constructed by DTI and T1 features, which exhibited the anatomical framework of intrinsic structures, depicted in the first column of Fig. 2A and B. Meanwhile, MCNs were constructed by resting-state EEG features (E-MCNs), showing the interregional interactions of functional activity (the second column of Fig. 2A and B). Although MRI reflects highly spatial-resolved brain information and EEG captures the time-resolved functional activity, the fusion of multimodal features is still unexplored when investigating the CNs. As such, based on the structural framework, the resting-state fMRI or EEG features were merged to testify how functional dynamics covary with its morphological substrates, and the structural–functional MCNs (SF-MCNs) and structural–EEG MCNs (SE-MCNs) were accordingly constructed (the third and fourth columns of Fig. 2A and B, respectively). In addition, the combination of the fMRI and EEG features further constitutes the functional–EEG MCNs (FE-MCNs), which preliminarily capture the intramodularity covarying of the visual, somatomotor, and limbic networks (the fifth column of Fig. 2A and B). Importantly, as displayed in the sixth column, the integration of the structural, functional MRI, and EEG features, i.e., the structural–functional–EEG MCNs (SFE-MCNs), eventually presents the strengthened modular covarying within the visual, somatomotor, and limbic networks.

Since hierarchical modularity is generally considered as an organizing principle of the human brain [22], the network modularity was computed and compared among various MCNs. As expected, the network modularity was consistently increased with the fusion of more brain modalities, and especially, the SFE-MCN modularity is significantly and consistently increased when compared to that of S-MCN, SF-MCN, E-MCN, and SE-MCN in both cohorts (*P* < 0.05; Fig. 2C and D). Although no significant difference was identified between the SFE-MCN and FE-MCN modularity (*P* = 0.12), enhanced network modularity of SFE-MCN compared with the FE-MCN can still be observed (Fig. 2C and D). More importantly, the modular parameter of SFE-MCN was significantly different between resting- and task-state in the discovery cohort (Fig. 2E), as well as between MDDs and HCs in the validation cohort (Fig. 2F). No such interstate and intergroup differences are identified for S-MCN, E-MCN, SF-MCN, SE-MCN, and FE-MCN. In addition, taking the task behaviors into account, we then explored the relationships between the task MCN properties [i.e., the characteristic path length (*L*), global efficiency (*Ge*), local efficiency (*Le*), and clustering coefficient (*C*)] and risky performance during the gambling task for the discovery cohort (Table S4 and Supplementary Results 1). Specifically, for S-MCN, E-MCN, and SF-MCN, no correlations between network properties and risky rates were identified (*P* > 0.05). Interestingly, although the SE-MCN, FE-MCN, and SFE-MCN properties were identified to be significantly related to the risky performance (*P* < 0.05), SFE-MCN exhibited the highest correlation coefficients (Table S4 and Fig. 2G; *C*: *r* = 0.51, *P* = 0.01; *L*: *r* = −0.53, *P* = 0.01; *Ge*: *r* = 0.54, *P* < 0.01; *Le*: *r* = 0.52, *P* = 0.01), suggesting the highest potential and sensitivity of SFE-MCN in reflecting individual cognitive performance. Namely, the inclusion of EEG attributes with high temporal precision effectively increases the sensitivity of MCN in detecting cognition-related neural activity. Likewise, for the MDD patients in the validation cohort, the relationships bet-ween MCN properties and the Hamilton Depression Rating Scale (HAMD) were also assessed in Table S5. In detail, no significant correlations were identified between clinical scales and properties of S-MCN, E-MCN, SF-MCN, SE-MCN, or FE-MCN (*P* > 0.05). Only the *L* (*r* = 0.18, *P* = 0.01) of SFE-MCN exhibited a significant positive correlation with the HAMD scores, and the *Ge* (*r* = −0.21, *P* < 0.01), *Le* (*r* = −0.19, *P* = 0.01), and *C* (*r* = −0.18, *P* = 0.01) of SFE-MCN exhibited negative relationships (Fig. 2H), which is helpful to track the disease severity of depression patients.

**Fig. 3.**
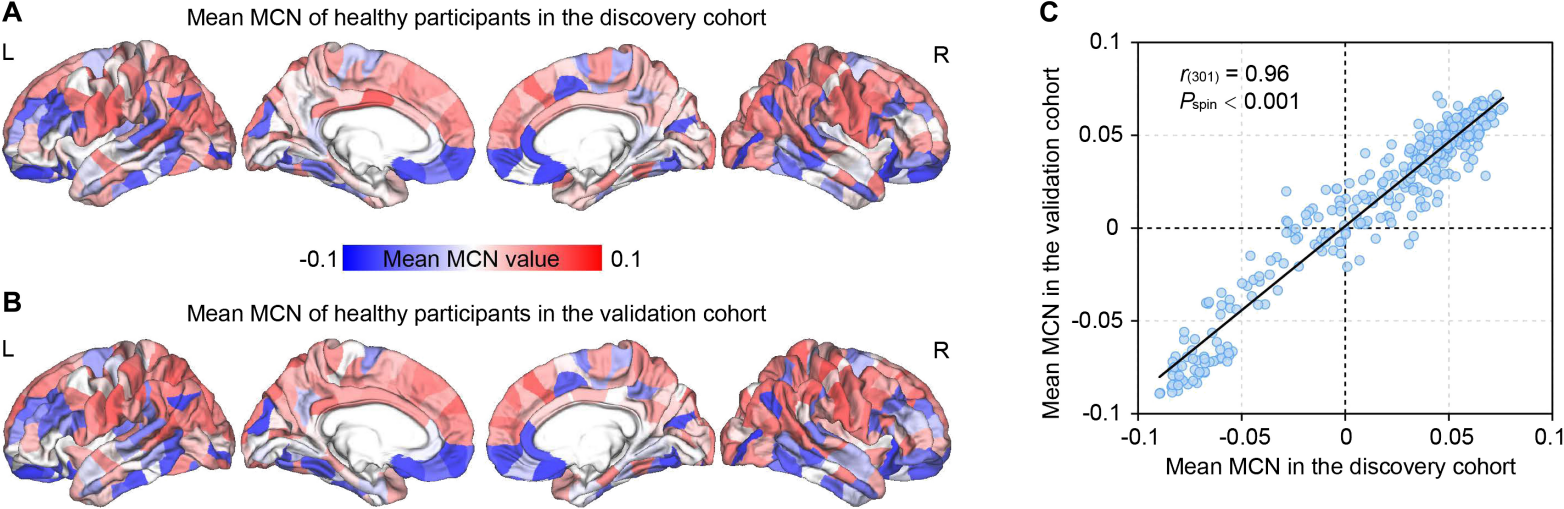
Replicable resting-state SFE-MCN pattern in healthy people. (A) Mean regional SFE-MCN averaged over healthy people in the discovery cohort. (B) Mean regional SFE-MCN averaged over healthy people in the validation cohort. (C) Similarity analysis of regional SFE-MCN between the 2 independent cohorts [*r*_(301)_ = 0.96, *P*_spin_ < 0.001].

Furthermore, the sum of the weighted linkages between a specific area and its connections to others was calculated and defined as the regional MCN. Then, the grand average regional MCNs of the discovery and validation cohort were separately calculated, and Pearson’s correlation was adopted to probe the spatial similarity of regional MCN between the 2 independent cohorts. From Table S6, we found that the regional MCN replicability between the 2 cohorts was increased, along with the addition of information from more modalities, and especially, among these MCNs, the spatial patterns of the regional SFE-MCN showed the strongest correlation between the 2 groups of healthy participants [*r*_(301)_ = 0.96, *P*_spin_ < 0.001; the spatial autocorrelation was corrected by a “spin”-based method [23]]. Figure 3 then illustrates the grand average regional SFE-MCN topologies of healthy participants from the discovery (Fig. 3A) and validation cohort (Fig. 3B), as well as the similarity analysis of regional SFE-MCN between the 2 cohorts (Fig. 3C). Overall, given the high sensitivity and replicability of SFE-MCN in detecting the structural–functional covarying of the human brain, our subsequent analyses were primarily performed on SFE-MCNs to further uncover the cognition- and disease-specific gene expressions in the discovery and validation cohort, respectively. Hence, MCN refers to SFE-MCN in the following results, unless stated otherwise.

### Cognitive task-evoked changes in MCN

**Fig. 4.**
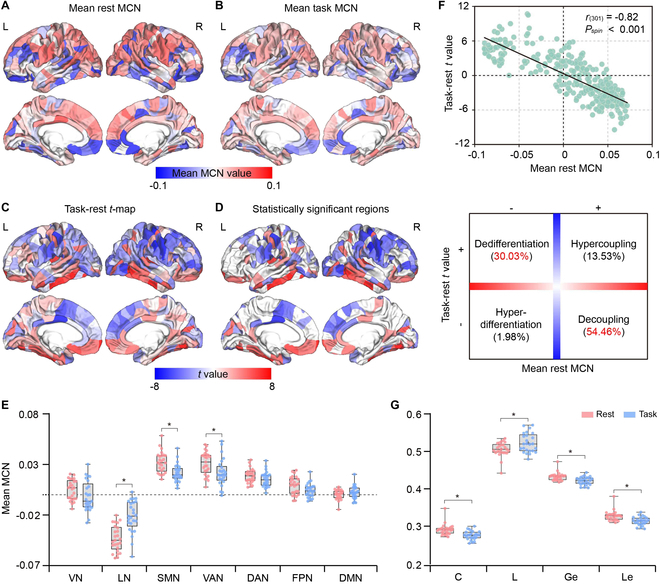
Task-rest differences of regional MCN. (A) Mean regional MCN patterns of the resting state. (B) Mean regional MCN patterns of the task state. (C) Task-rest comparison (*t* map) of regional MCN. (D) Significant differences in cortical areas (*P*_FDR_ < 0.05). (E) Distribution of task-rest differences in Yeo 7 functional networks. VAN, ventral attention network; DAN, dorsal attention network; FPN, frontoparietal network; DMN, default mode network; SMN, somatomotor network; LN, limbic network; VN, visual network. The asterisks indicate significant differences between the 2 states (SMN: *P*_FDR_ < 0.01; VAN: *P*_FDR_ < 0.01; LN: *P*_FDR_ = 0.01). (F) Correlations between the task-rest *t* map and mean regional MCN (upper row) [*r*_(301)_ = −0.82, *P*_spin_ < 0.001]. Most cortical regions exhibited decoupling (54.46%) and dedifferentiation (30.03%) in individuals playing gambling tasks (bottom row). (G) Network properties of MCN. The asterisks denote the significant differences between the 2 states.

We then mapped the resting- and task-state regional MCNs of the discovery cohort (Fig. 4A and B), based on which the task-rest *t* map was obtained by performing a paired-sample *t* test (Fig. 4C). Negative *t* values indicate attenuated structural–functional covarying in participants involved in the gambling task in contrast to a rest condition, and vice versa. As exhibited in Fig. 4D, when shifting from baseline to fulfill the demanding needs of the gambling task, significantly decreasing frontal covarying, as well as increasing parietal and temporal covarying were identified. Meanwhile, the cortical areas were further reallocated to the Yeo 7 functional networks. Interestingly, when reconfiguring from rest to the gambling task, the brain exhibited significantly increased structural–functional covarying in Yeo limbic network but decreased covarying in Yeo somatomotor and dorsal attention networks (Fig. 4E). Further investigation found the asymmetric but moderately correlated (*r* = 0.43, *P* < 0.001) regional MCN alterations between the left and right hemispheres (Fig. S1A and Supplementary Results 3).

In addition, the task-rest *t* map has a significant spatial correlation with the resting-state regional MCN [*r*_(301)_ = −0.82, *P*_spin_ < 0.001; Fig. 4F], suggesting that brain areas with stronger structural–functional covarying exhibit stable baseline configurations, while others tend to flexibly shift to fulfill the needs of the task performance. Negative *t* values and positive mean MCN jointly represent stable baseline patterns in rest relative to the task, which was exhibited in 54.46% decoupling of the brain regions, whereas 30.03% had positive *t* values and negative mean MCN, indicating dedifferentiation when individuals were involved in the gambling task relative to the rest. Also, the corresponding MCN properties between the 2 states were further compared. In Fig. 4G, during the gambling task, decreased *C*, *Ge*, and *Le* and prolonged *L*, deviating from the resting state, were identified, coinciding with the extensive attenuated regional MCN in Fig. 4C.

### Cortical gene expression related to cognitive task-evoked changes in regional MCN

**Fig. 5.**
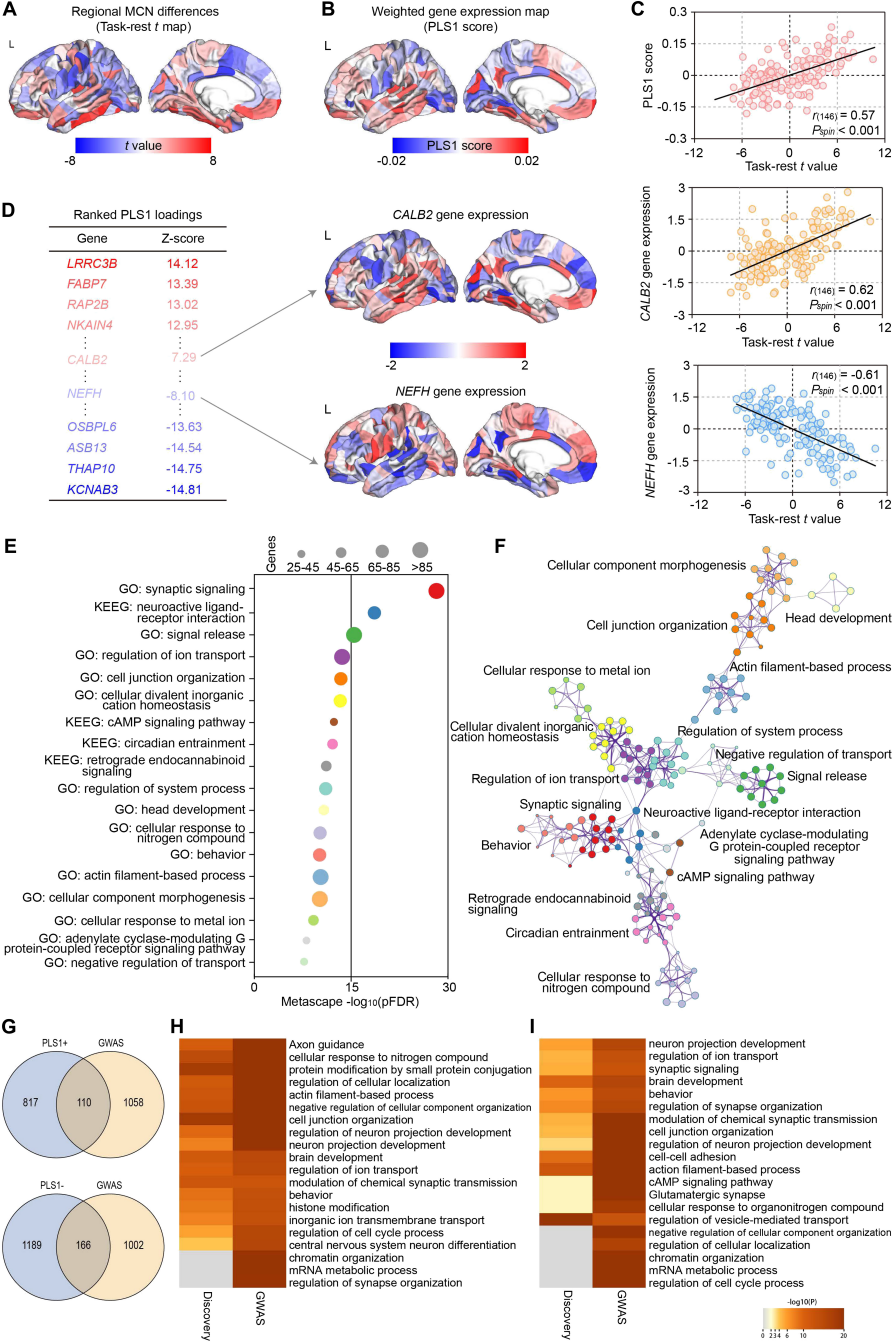
Transcriptomic decoding of the gambling-evoked structural–functional covarying differences. (A) Task-rest comparison (*t* map) of regional MCN in the left hemisphere. (B) Cortical map of regional PLS1 scores. (C) Correlations between regional MCN alterations and PLS1 scores [*r*_(146)_ = 0.57, *P*_spin_ < 0.001]. (D) Genes with strong positive weights on PLS1 related positively to task-evoked differences in regional MCN (e.g., *CALB2*: *r* = 0.62, *P*_spin_ < 0.001), and vice versa (e.g., *NEFH*: *r* = −0.61, *P*_spin_ < 0.001). (E) Functional enrichment analysis of PLS1^+^ genes (*P*_FDR_ < 0.01). (F) Metascape enrichment network visualizes the inter- and intracluster similarities of enriched terms. (G) Overlapped genes between the PLS1^+^/PLS1^−^ gene list and that from GWAS. (H) Overlapped ontology terms between the PLS1^+^ genes and that from GWAS. (I) Overlapped ontology terms between the PLS1^−^ genes and that from GWAS.

Thereafter, to investigate the relationship between gambling-evoked MCN alterations (Fig. 5A) and transcriptomic variations, the AHBA transcriptomic dataset and PLS regression were applied. Thereinto, the first PLS component (PLS1) represented the cortical expression map that is strongest related to the task-rest MCN differences (Fig. 5B), and the corresponding PLS1 could explain 32% of the variance in the task-rest MCN differences (*P*_perm_ < 0.001). No statistical significance was achieved for the other PLS components (*P*_perm_ > 0.05; Table S7 and Supplementary Results 4); therefore, only PLS1 was included in the subsequent analysis. As exhibited in Fig. 5C, the PLS1 weighted gene expression map was found to be spatially correlated with the task-rest *t* map [*r*_(146)_ = 0.57, *P*_spin_ < 0.001]. Furthermore, applying univariate one-sample *Z* tests, the candidate gene list was ranked with their normalized weights of PLS1, and 927 positively (PLS1^+^: *Z* > 5) and 1,355 negatively (PLS1^−^: *Z* < −5) weighted genes were identified. Moreover, the gene expression level of each PLS1^+^ or PLS1^−^ gene was significantly related to the task-rest MCN *t* value (Fig. 5D). Afterward, applying Metascape analysis [24], the Kyoto Encyclopedia of Genes and Genomes (KEGG) pathways and Gene Ontology (GO) biological processes were aligned with the PLS1^+^ and PLS1^−^ gene list, respectively. As provided in Fig. 5E and F, for the PLS1^+^ gene list, after discarding discrete enrichment clusters and correcting for enrichment terms (*P*_FDR_ < 0.01), the top 14 GO biological processes including “synaptic signaling,” “signal release,” and “regulation of ion transport” were identified, as well as 4 KEGG pathways (e.g., “cAMP signaling pathway” and “neuroactive ligand-receptor interaction”). In addition, the PLS1^−^ gene list was enriched for 5 GO biological processes including the “inorganic ion transmembrane transport” and “action potential,” together with 3 KEGG pathways, such as the “MAPK signaling pathway” (Fig. S2 and Supplementary Results 5).

To further characterize these candidate genes for the cognitive task, we compared the PLS1 results with a previous meta-analysis of genome-wide association study (GWAS) of educational attainment and cognitive performance [25]. Interestingly, the PLS1^+^ (PLS1^−^) genes overlapped significantly with genes from GWAS (*P*_perm_ < 0.05; Fig. 5G). Then, a multi-gene list meta-analysis between GWAS genes and the PLS1^+^ (PLS1^−^) gene list was performed. Consistently, we found that 17 of 20 enrichment pathways from GWAS overlapped with the PLS1^+^ genes, including “synaptic signaling,” “brain development,” “regulation of ion transport,” and “behavior” (Fig. 5H), while 15 of 20 enrichment pathways from GWAS were overlapped with the PLS1^−^ genes, including “regulation of neuron projection development” and “cell junction organization” (Fig. 5I). The detailed distribution of overlapped ontology terms is exhibited in Figs. S3 and S4.

### Cellular characterization of the cognitive task-related genes

**Fig. 6.**
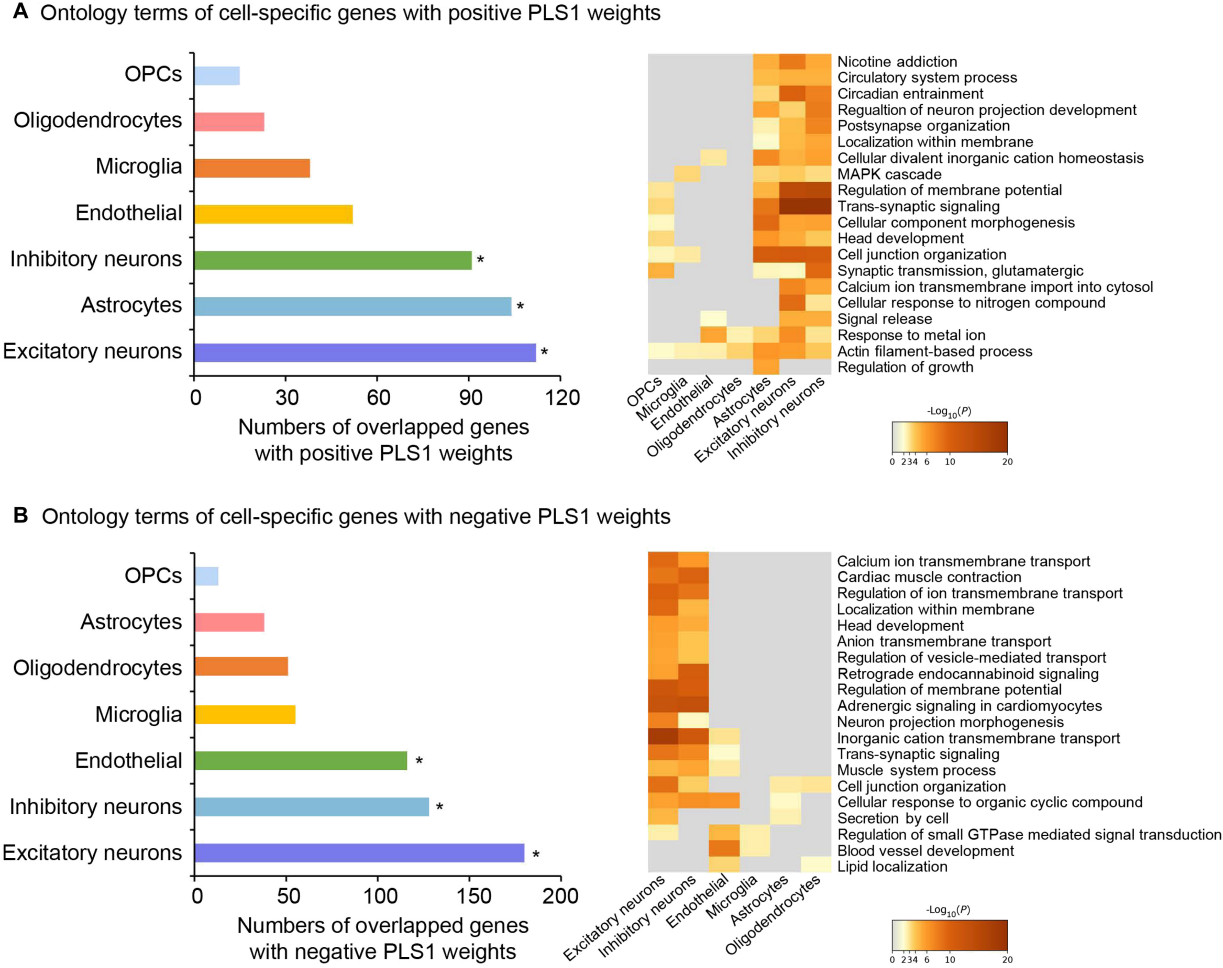
Cell type specificity of gambling-related MCN alterations. Enrichment analysis of cell-specific gene lists with PLS1^+^ (A) and PLS1^−^ (B) weights. Left panel represents the number of overlapping genes in each cell type. *P_perm_ < 0.05, FDR-corrected. Right panel represents the enrichment pathways for each cell type.

Using previously defined cell class gene sets [19], we found that the PLS1^+^ and PLS1^−^ genes were expressed primarily in both excitatory (PLS1^+^: 112 genes, *P*_perm_ = 0.01; PLS1^−^: 180 genes, *P*_perm_ < 0.01) and inhibitory neurons (PLS1^+^: 91 genes, *P*_perm_ < 0.01; PLS1^−^: 128 genes, *P*_perm_ < 0.01; Fig. 6). Likewise, genes specific to astrocytes (PLS1^+^: 104 genes, *P*_perm_ < 0.01) and endothelial cells (PLS1^−^: 116 genes, *P*_perm_ = 0.01) were overrepresented in the PLS1^+^ and PLS1^−^ genes, respectively (the first column of Fig. 6A and B). Consistently, enrichment analysis of cell type-specific gene lists verified that alterations of MCN were significantly enriched for biological processes related to neuronal cells (the second column of Fig. 6A and B).

### MDD-MCN changes correlated with transcriptomic variations from the validation cohort

**Fig. 7.**
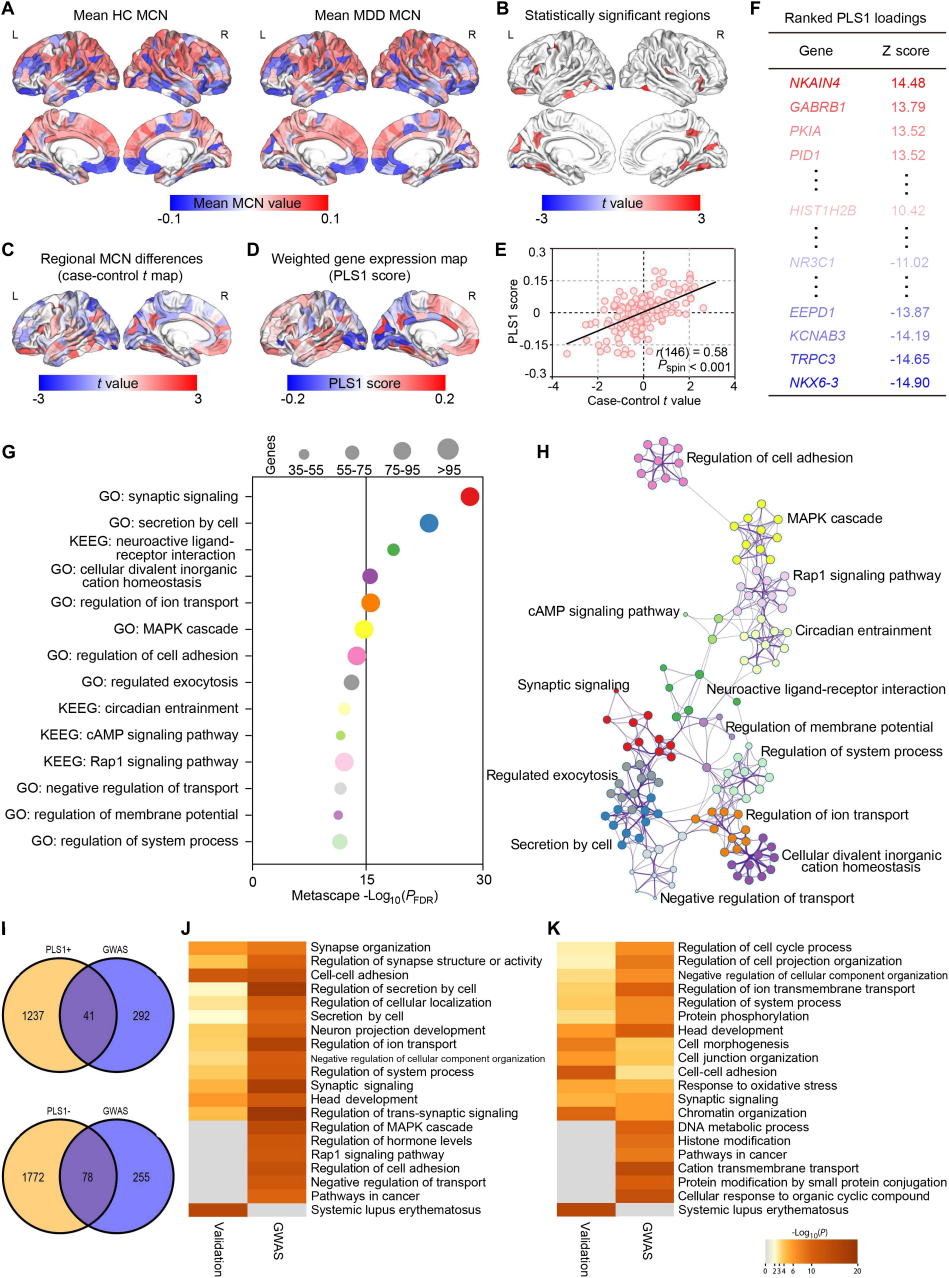
Transcriptomic decoding of the MDD-related structural–functional covarying differences. (A) Mean MCN pattern of HCs and MDDs. (B) Significant differences in cortical areas between MDDs and HCs (*P*_FDR_ < 0.05). (C) Case–control comparison (*t* map) of regional MCN. (D) Cortical map of regional PLS1 scores. (E) Correlations between regional alterations in MCN regional and PLS1 scores [*r*_(148)_ = 0.58, *P*_spin_ < 0.001]. (F) Ranked PLS1 loading. (G) Functional enrichment analysis of PLS1^+^ genes (*P*_FDR_ < 0.01). (H) Metascape enrichment network visualizes the inter- and intracluster similarities of enriched terms. (I) Overlapped genes between the PLS1^+^/PLS1^−^ genes and that from GWAS. (J) Overlapped ontology terms between the PLS1^+^ genes and that from GWAS. (K) Overlapped ontology terms between the PLS1^−^ genes and that from GWAS.

Here, not only within the HCs, MCNs of MDD patients were further constructed, subserving as the validation cohort. Figure 7A exhibits the grand average cortical map of regional MCN in HCs and MDDs. We then mapped the case–control *t* statistics and the statistically significant differences in regional MCN between MDDs and HCs in Fig. 7C and B, respectively. Thereinto, negative *t* denotes decreased structural–functional covarying in MDDs in comparison with HCs, and vice versa. Specifically, the increased regional MCN is mainly located in the brain areas such as inferotemporal and insula for patients with MDD, while the decreased regional MCN was only found in the lateral occipital area (*P*_FDR_ < 0.05; Fig. 7B). Likewise, for the validation cohort, the regional MCN alterations were correlated (*r* = 0.52, *P* < 0.001; Fig. S1B and Supplementary Results 3) but asymmetric between the 2 hemispheres (*P* < 0.05). When using the spatial pattern of the network (SPN) features extracted from MCNs [26], an accuracy of 90.71% could be achieved to recognize MDD patients from healthy populations, which confirmed that MCN can facilitate the individual-level pattern recognition and further validated the capacity of MCN in capturing the MDD-specific structural–functional abnormalities.

Thereafter, PLS regression was also applied, and the corresponding results showed that PLS1 (Fig. 7D) can explain 34% of the variance in the case–control MCN differences, significantly more than expected by chance (*P*_perm_ < 0.001). However, the other PLS components were not statistically significant by the permutation testing (*P*_perm_ > 0.05; Table S8 and Supplementary Results 4), and thus unconsidered for the following analysis. Particularly, the weights of PLS1 gene expression were significantly positively correlated with these MCN differences [*r*_(146)_ = 0.58, *P*_spin_ < 0.001; Fig. 7E], which were further ranked by a univariate one-sample *Z* test (Fig. 7F). Metascape was then performed to align the GO biological processes and KEGG pathways with the PLS1^+^ or PLS1^−^ gene list. For the PLS1^+^ gene list (*Z* > 5), the enriched biological processes included “synaptic signaling,” “secretion by cell,” and “cellular divalent inorganic cation homeostasis” (Fig. 7G and H). Meanwhile, we found 4 significantly enriched KEGG pathways, such as “circadian entrainment” and “neuroactive ligand-receptor interaction.” Additionally, Metascape analysis of the PLS1^−^ gene list (*Z* < −5) identified 7 GO biological processes such as “regulation of system process,” “regulation of membrane potential,” and “trans-synaptic signaling,” while no KEGG pathways were enriched (Supplementary Results 7 and Fig. S5).

Furthermore, based on 3 recent GWAS research [27–29], genes relating to the MDD phenotype were compiled. As exhibited in Fig. 7I, we found that 119 of 333 (35.75%) genes of GWAS significantly overlapped with the MDD-related PLS1^+^ and PLS1^−^ genes (*P*_perm_ < 0.05). Moreover, enrichment pathways of the PLS1^+^ (PLS1^−^) genes comprise 13 of 19 pathways of genes from the MDD GWAS (Fig. 7J and K), which included “head development,” “synaptic signaling,” and “regulation of ion transport.” The detailed distribution of overlapped ontology terms is exhibited in Figs. S6 and S7. Besides, we found 12 well-known candidate genes for MDD in the PLS1 genes [30], among which 10 genes were significantly associated with regional alterations in MCN, including *HTR1A*, *CNR1*, *SST*, *TAC1*, *PDE1A*, *MAOA*, *CUX2*, *CHRM2*, *HTR5A*, and *ARRA2A* (*P*_FDR_ < 0.05; Supplementary Results 9 and Fig. S8).

Afterward, we found that the PLS1^+^ genes were expressed primarily in astrocytes (156 genes, *P*_perm_ < 0.01) and microglia (113 genes, *P*_perm_ < 0.01) (Fig. S9A), while genes specific to excitatory neurons (228 genes, *P*_perm_ < 0.01) and inhibitory neurons (161 genes, *P*_perm_ < 0.01) were overrepresented in the PLS1^−^ gene set (Fig. S9B). Consistently, Metascape analysis of cell type-specific genes uncovered that changes in MDD-MCN were significantly enriched for synapse function and neuroinflammation-related processes in astrocytes, microglia, and neurons, such as “synapse organization,” “signaling pathway,” and “synaptic signaling.”

### Reproducibility of SFE-MCN superiority and corresponding transcriptomic profile

We further validated the superiority and repeatability of SFE-MCN, as well as transcriptional profiles linked to regional MCN alterations using a different brain atlas, i.e., the human Brainnetome atlas [31]. First, the identically derived S-MCN, E-MCN, SF-MCN, SE-MCN, FE-MCN, and SFE-MCN were assigned to each of the Yeo 7 functional networks (Fig. S10A and Supplementary Results 11). Results show that the spatial patterns of MCN constructed by the Brainnetome atlas were similar to those constructed by the Desikan–Killiany (D-K) atlas (Fig. 2A and B); particularly, with the addition of more brain modalities, MCN further presents the visually strengthened modular covarying within the visual, somatomotor, and limbic networks. Quantification analysis also verified that the network modularity was consistently increased with the fusion of more modalities; especially, the modularity of SFE-MCN is significantly larger than that of S-MCN, SF-MCN, E-MCN, and SE-MCN (*P* < 0.05; Fig. S10B). Besides, significantly increased modular parameters of the resting state in contrast to the task state were found for both SF-MCNs and SFE-MCNs (*P* < 0.05; Fig. S10C), whose network properties were further found to be significantly correlated with the risky rates of the gambling task (*P* < 0.05; Table S9 and Fig. S10D). Namely, using a different brain atlas, we complementally validate the superiority and repeatability of SFE-MCN in capturing the cognition-specific structural–functional covarying of the human brain.

Thereafter, leveraging the identical strategy, the task-rest *t* map of MCN was obtained based on the Brainnetome atlas, and PLS regression was performed to map these MCN variations to anatomically patterned gene expression from the AHBA. Results show that PLS1 could explain 32% of the variance in the task-rest MCN differences (*P*_perm_ = 0.01), close to that based on the D-K atlas. Thereinto, we found that 837 PLS1^+^ (*Z* > 5) genes and 1,140 PLS1^−^ (*Z* < −5) genes were significantly overexpressed in cortical regions, consisting of 1,977 regional MCN gene list differences. To further validate the gene list obtained by MCN analysis, a multi-gene list meta-analysis [24] was conducted between the PLS1^+^ (PLS1^−^) gene lists obtained from the D-K and Brainnetome atlas. Notably, the gene lists derived from the 2 atlases were highly overlapped (left panel of Fig. S11 and Supplementary Results 11). More importantly, the enrichment pathways were also highly overlapped with only one pathway not matched (right panel of Fig. S11 and Supplementary Results 11), further validating the generalized relationship between gene expression and the regional MCN alterations.

## Discussion

Until recently, the majority of investigations in neuroimaging using individual CN have left the multimodal information unfused, although further integration would allow a more comprehensive understanding of the brain’s structural and functional interactions. Hence, rather than using only unimodal features, our MCNs combine information from multiple structural and functional measurements in a single subject to assess the interregional covarying patterns. In addition, the link between MCN and transcriptional data was also examined to delineate the relationship between transcriptomic profile and structural–functional covarying in specific cognitive processes and psychiatric patients. To our best knowledge, no study to date has explored the molecular basis of cognition- and disease-specific structural–functional covarying in the human brain.

### Effectiveness and repeatability of MCN

We first validated the effectiveness of the MCN method in 2 independent MRI-EEG cohorts. For comparison, in addition to the resting-state MCNs constructed by integrating all MRI-EEG modalities (SFE-MCN), CNs constructed by structural MRI (S-MCN) or EEG alone (E-MCN), and the pairwise combination of the structural, functional MRI, and EEG features (i.e., SF-MCN, SE-MCN, and FE-MCN) were also investigated. As illustrated, when functional features were merged into the CNs’ construction, corresponding state-specific modules, such as YEO-visual, limbic, and somatomotor networks [32–34], were highlighted. These hierarchical modules appeared increasingly prominent when features of all modalities were further fused. Quantitative analysis consistently confirmed that the modularity measurement of SFE-MCNs was consistently increased when compared to that of S-MCN, SF-MCN, E-MCN, SE-MCN, and FE-MCN in both cohorts. This coincided with our hypothesis since the inclusion of functional attributes would increase the sensitivity of MCN in detecting brain neural activity based on the intrinsic morphological framework. Especially EEG provides complementary temporal resolution to MRI for continuous reading out of the fine-grained details of cognitive processes [6].

Modularity facilitates the dynamic shifting between brain segregation and integration and is constantly adjusted to fulfill the needs of cognition. As demonstrated, modularity will decrease, along with increased cognitive demands [35]. This was consistently observed in our current results as the modularity of the task-state SFE-MCN was significantly decreased than that of the rest. Likewise, in comparison with HCs, the SFE-MCN modularity of MDDs was significantly attenuated, aligning with the “disconnectivity” commonly recognized in MDD patients [36]. No such interstate and intergroup differences are identified in S-MCN, E-MCN, SF-MCN, SE-MCN, and FE-MCN. Additionally, considering that the gambling task in the discovery cohort requires the maximized profit, the risky decision, which was more conducive to achieving this goal, was quantitatively investigated to explore its relationship with MCN properties. Generally, the larger *C*, *Ge*, and *Le*, as well as a smaller *L*, index higher information processing efficiency of the human brain [37]. As such, when SFE-MCN is operating efficiently, it will result in a higher risky rate to achieve the goal of benefit maximization. For the validation cohort, the SFE-MCN properties of MDD were found to be correlated with the corresponding HAMD scores, which have the potential to track the severity of depression in patients. However, these correlations were relatively weak or could not be achieved by the limited information provided merely by S-MCN, E-MCN, SF-MCN, SE-MCN, and FE-MCN. Of note, the superiority of SFE-MCN was also validated using the Brainnetome atlas. In addition, given the highly consistent SFE-MCN patterns among different healthy individuals from both cohorts, SFE-MCNs are proven to be replicable and have great potential for capturing the cognition- or disease-specific structural–functional covarying in the human brain. Hence, SFE-MCN was further adopted to uncover the cognition- and disease-specific gene expressions in the discovery and validation cohort, respectively.

### Cognition-specific MCN changes and potential links with transcriptomic patterns

As we know, decision-making relies on a complex set of cognitive processes that are orchestrated across various brain modules to achieve a satisfying gain. At the task onset, visual information is quickly transmitted and processed in the occipital and parietal cortex; then, except for the areas responsible for primary sensory processing, neuronal ensembles of the frontal and parietal cortex that are associated with attention and working memory [38,39] are activated to establish strong interactions to other regions for decision making. Therefore, in contrast to the resting state that functions as a highly integrated interoceptive state, the modular covarying within the somatomotor and dorsal attention networks experienced significant suppression, which further establish extensive interaction with other functional subnetworks. Concurrently, the flexibly adaptive behavior during the task will involve a host of brain areas of the limbic system [40,41], further resulting in stronger structural–functional covarying in the limbic network.

Alterations in brain connectivity may owe to numerous factors, e.g., neuronal changes at the genetic transcriptomic, molecular, and cellular levels [42]. Based on the multivariate PLS approach, we discovered that distributions of whole-brain weighted gene expression were significantly colocalized with gambling-evoked structural–functional alterations. Furthermore, the PLS1^+^ and PLS1^−^ genes may play essential roles in the operation of gambling-related high-level cognitive functions as their expression levels were either positively or negatively associated with the task-rest MCN differences. For example, the positively weighted gene *CALB2* is of great significance for synaptic transmission, forming the basis of memory and learning [43]. The impact of *NEFL* on synapses and plasticity has also been suggested [44]. The down-regulation of *NEFH* in alcoholics may be related to the changed behavioral responses usually observed in addiction [45], which is consistent with the potential addictive behavior when an individual is involved in a gambling condition.

The topologically clustered interaction networks derived from these positively and negatively weighted genes were enriched for a series of KEGG pathways and GO biological processes highly relevant to brain cognition. Here, the observed KEGG pathways including “neuroactive ligand-receptor interaction” and “retrograde endocannabinoid signaling” may regulate multiple neural or synaptic neurotransmission functions, such as pain, motor control, and cognition [46,47]. Especially, the “synaptic signaling” process showing the highest Metascape value was reported to influence synaptic maturation and stability [48]. In addition, ion transport is of great significance for the stability of brain neural circuits [49], and the expression profile of the genes related to potassium ion transport may be involved in the spatial heterogeneity of module dynamics [50]. As such, the enrichment of “inorganic ion transmembrane transport,” “regulation of transmembrane transport,” and “regulation of ion transport” pathways in our study may thereby mediate the structural–functional modular configuration when involved in the gambling task. More importantly, significantly overlapped candidate genes and largely shared pathways with GWAS of educational attainment and cognitive performance provide further support for the sensitivity and reliability of candidate genes and pathways obtained by the PLS method in our discovery cohort.

We further investigated how cell type-specific manners influence cognition-specific structural–functional covarying. As illustrated, excitatory and inhibitory neurons occupy a high proportion under the gene expression profiles obtained by the PLS, followed by astrocytes and endothelial cells. Generally, the functional and structural plasticity of the brain needs selective and organized modulation of inhibitory and excitatory synapses to promote the representation of behaviorally essential sensory stimuli while maintaining the excitability of the overall network [51]. In parallel, the excitatory/inhibitory balance is considered to be a potential mechanism that plays an essential role in mental processes and the formation of the higher cognitions evoked by tasks [52]. Besides, we found that the regulation of gene expression during the gambling task was also related to astrocytes, which was consistent with a previous study reporting that sensory information processing is associated with the coordinated activity of cortical neurons and astrocytes [53]. Moreover, astrocytes are adept at dynamically controlling glutamate at specific synapses, further influencing postsynaptic neuronal activity and potentially driving individual task behavior [54]. Finally, leveraging multimodal structural and functional features, we found that most enriched pathways were associated with excitatory/inhibitory neurons, astrocytes, and endothelial cells.

### MDD-related MCN changes and potential links with brain transcriptomic patterns

The disease-specific structural–functional abnormality and its relationship with transcriptomic profile were also explored for MDD patients in the validation cohort. First, we found that patients with MDD exhibited significantly enhanced regional MCNs in contrast to that of HCs. Considering that MDD has been generally accepted as a “dysconnectivity” brain disorder from both structural and functional dimensions, the synchronous attenuation of anatomical morphology and functional activity thereby experienced significant enhancement in brain areas including the inferior temporal, fusiform, and insula. Furthermore, a previous study has suggested that MDD could be regarded as a network-based disease with consistent alterations in functional connection modes [36], and more importantly, these functional alterations are associated specifically with structural dysconnectivity in depression patients [55], which echoes our current result.

Thereafter, PLS was applied, and the strong correlations detected between MDD-related gene expression and regional MCN alterations demonstrated the sensitivity of MCNs in detecting MDD-specific molecular variations. Then, the PLS1^+^ and PLS1^−^ genes were significantly enriched for several KEGG pathways and GO biological processes. Thereinto, the 4 KEEG pathways, i.e., “Rap1 signaling pathway,” “cAMP signaling pathway,” “circadian entrainment,” and “neuroactive ligand-receptor interaction,” have also been proposed in previous studies about MDD-related pathogenic mechanisms [15,56]. As for the GO biological processes, the “synaptic signaling” pathway influencing synaptic stability and maturation [48] was identified with the highest Metascape value. Postmortem research has shown that MDD patients have fewer synapses and lower expression of genes correlated with synaptic function [57]. The preclinical study also demonstrated lower synaptic density in the experimental model of MDD, specifically in the hippocampal regions and prefrontal cortex [48]. Importantly, consistent with the results of MDD-related GWAS, we identified similar synapse-related ontology terms, further validating the sensitivity and reliability of genes and pathways captured by the PLS method in the validation cohort. Hence, synaptic deficits in MDD could be a target for clinical treatment. Future work may focus on investigating the in vivo synaptogenesis effects of rapid-acting antidepressants in humans, and alternative therapies (e.g., exercise and mindfulness) that can strengthen synaptic linkages and stabilize the brain network effects.

We finally explored whether the genes of MDD-related structural–functional alteration could be translated into alterations of specific cell types. Our result showed that astrocytes exhibited the greatest proportion among the gene expression profiles obtained by PLS1^+^. As reported previously, alterations in the density of glial cells have been found in MDD [58]. Astrocytes secrete signaling molecules that promote synapse formation and function and release other bioactive substances to regulate synaptic transmission [59], therefore implicating a novel and active role for astrocytes in promoting synapse formation and function. Strikingly, this was quite consistent with the above discussion that the synaptic deficit has been identified as a critical pathological trait in MDD, and astrocytes have been regarded as key mediators of neuroinflammation involved in MDD pathogenesis [60]. Besides, the dysregulation of gene expression in MDD was also associated with microglia and excitatory/inhibitory neurons. In essence, the activated microglia phenotype is associated with depression, contributing to the precise regulation of microglia to investigate the therapeutic mechanism of depression [61], and neurons are also concerned as the target cell types in MDD pathophysiology [62]. Thus, understanding the role of these cells in synaptic function and inflammation promises to be a fruitful area for future investigation in understanding how to build targeted therapies for MDD patients.

One limitation of our current work was that due to only 2 right hemispherical data being available in the AHBA dataset, the relationship between genes and regional MCN alterations cannot be accounted for in the entire brain. Although we have identified a relatively high correlation between the left and right regional MCN alterations, some right hemisphere-specific genes are still inevitably missed due to the hemispherical asymmetry. In future work, it will be valuable to profile brain-wide MCN alterations by using more fully characterized transcriptomes with the construction of more whole-brain gene expression atlases.

## Materials and Methods

### Experimental design

With the approval of the Ethics Committee of the University of Electronic Science and Technology of China, 25 right-handed participants (4 females, aged 25.64 ± 1.65 years) were enrolled in the discovery cohort. None of them had ever used prescribed medication over an extended period or had any family or personal history of neurological or psychiatric diseases. Written informed consent was accordingly obtained from all the subjects before the multimodal data collection. Participants in the discovery cohort performed a classical gambling experiment according to a standard protocol [63], during which resting- and task-state fMRI/EEG datasets were collected, while the dMRI and T1 images were scanned before and after the experiment, respectively. The gambling experiment included a 5-min resting state and 8 blocks of gambling tasks, and in each block, 40 trials were designed. In each trial, to notify the participant to focus on the computer screen, a thin cross sign was presented on the screen and lasted 500 ms; 2 adjacent squares presented 2,000 ms, writing “¥25” or “¥5.” In the following 2,000 ms, the participant was requested to press the corresponding button to select one bet, and a white screen lasting 500 ms was set for a short rest. Afterward, the chosen square turned red or green to signal a monetary loss or a win (i.e., green for a win and red for a loss), which lasted 2,000 ms. After finishing one loop per trial, the next trial would initiate immediately. The task took about 30 min in total for each participant.

The validation brain imaging and EEG datasets, plus demographic and clinical information, for 200 unmedicated MDD patients (119 females, aged 38.33 ± 13.50 years) and 40 sex- and age-matched HCs (22 females, aged 36.87 ± 14.81 years) were acquired from the EMBARC study. Details about the EMBARC study design can be found in [21]. Participants were enrolled at the University of Michigan (UM), the University of Texas Southwestern Medical Center (TX), Columbia University (CU), and Massachusetts General Hospital (MG), under the approval of the institutional review boards from the 4 sites. All of them provided written informed consent. HAMD was used to estimate depressive severity for MDD patients. During the experiment, EEG, T1, dMRI, and resting-state fMRI data were recorded for both the MDD patients and the HCs, and the participant was asked to relax their minds, inhibit eye movements, and keep still as possible. Seventeen MDDs and 5 HCs were discarded owing to large head motion (translation > 3.0 mm or rotation > 3.0°) and artifacts in EEG signals. Finally, 183 MDDs and 35 healthy individuals were included in the following analysis.

### Multimodal data acquisition

For the discovery cohort, MRI data were collected on a 3.0-T MRI scanner (GE Discovery MR750, USA) via a whole-head, 8-channel, standard coil. Details about the data collection parameters are presented in Table S1. During the gambling task, we used the E-prime 2.0 software (Psychology Software Tools Inc., USA) to exhibit the task procedures on a screen, from which participants can watch the experiment with a mirror attached to the head coil. Appling the ASA-Lab Amplifier (ANT Neuro), EEG datasets with 64 channels (Ag/AgCl electrodes, the 10–20 system) were collected at 500 Hz. Thereinto, AFz and FCz electrodes were applied as the ground and reference, respectively, and 2 extra electrodes were placed to monitor the electrooculogram and electrocardiogram, respectively. During online recording, the impedance of all electrodes remained consistently below 5 kΩ, and 0.01 to 70 Hz bandpass filtering was adopted.

MRI data in the validation cohort were recorded on a 3.0-T MRI scanner with different manufacturers. Detailed data collection parameters were presented in Table S2, and the EEG data were also recorded from CU, MG, TX, and UM. Specifically, at the UM, EEG data with a nose reference were recorded by a 32-bit NeuroScan Synamp (Compumedics) system and a Lycra stretch electrode cap (60 electrodes, 0.5 to 100 Hz bandpass filtering, 250 Hz sampling rate). Likewise, at the TX, EEG data with a nose reference were recorded by a 32-bit NeuroScan Synamp system and a Lycra stretch electrode cap (62 electrodes, DC-100 Hz bandpass filtering, 250 Hz sampling rate). As for the MG, EEG data were recorded by a Geodesic Net system with Cz as reference (129 electrodes, 0.01 to 100 Hz bandpass filtering, 250 Hz sampling rate). At the CU, EEG data with an active reference at electrode locations PPO2 and PPO1 were recorded by a 24-bit BioSemi system and a Lycra stretch electrode cap (72 electrodes, DC-251.3 Hz bandpass filtering, 256 Hz sampling rate).

### Data preprocessing

Using FreeSurfer (v6.0; http://surfer.nmr.mgh.harvard.edu/), the structural T1 images were first processed by following procedures: motion correction and averaging of T1 weighted images, removal of nonbrain tissue, automated Talairach transformation, segmentation of subcortical structures, intensity normalization, skull stripping, pial surface construction, and gray/white interface construction [64,65].

The FMRIB Software Library (FSL) [66] package was adopted for DTI preprocessing. By looping through individual images, we first performed a data quality assessment to check gross artifacts, such as interleave artifacts and signal dropouts caused by sudden participant motion. Then, images of each participant were corrected for eddy current-induced distortion and participant motion effect using the FMRIB Diffusion Toolbox. The first B0 image was adopted to produce the brain mask by the Brain Extraction Tool, and the linear least-squares fitting was applied to estimate the diffusion tensor model.

Using the Data Processing & Analysis of Brain Imaging (DPABI; http://rfmri.org/DPABI) toolkit, fMRI data were preprocessed by multiple steps. Concretely, the first 10 volumes were discarded, while the remaining ones were corrected for temporal differences and head motion. Thereafter, images were spatially normalized into the standard Montreal Neurological Institute (MNI) space, which was further resliced at a resolution of 2 × 2 × 2 mm^3^, spatially smoothed [6 mm full-width at half-maximum (FWHM)], and bandpass-filtered (0.01 to 0.1 Hz). Finally, regression of averaged cerebrospinal fluid signal, white matter signal, and 24 parameters acquired from the rigid-body head motion correction was performed.

The raw data were re-referenced to a neutral reference of the Reference Electrode Standardization Technique (REST) [67]. Independent component analysis (ICA) was applied to the re-referenced EEG signals to exclude ocular artifacts [68], followed by [0.5, 30] Hz offline bandpass filtering. Thereafter, for the discovery cohort, the resting-state data were segmented with 5-s length, while the task data were segmented by [−200, 1,800] ms (0 ms is the stimulus onset) with a [−200, 0] ms baseline correction. Segments with high-amplitude artifacts were discarded with a threshold of ±60 μV. Concerning the validation cohort, 10-s-length segmentation and ±100-μV artifact removal were conducted. Finally, to match the head model and high spatial resolution of MRI, standardized low-resolution electromagnetic tomography (sLORETA) [69] was performed to reconstruct the brain activities on the cortical layer.

### MCN construction

Based on the D-K atlas, the cortical surface of each participant was first divided into 308 spatially contiguous regions [20,70]. Meanwhile, the parcellated D-K atlas was expanded and interpolated to the corresponding dMRI and fMRI volumes, as well as to the participant’s EEG source space. Considering the delicate differences of the head model in MRI and EEG sources, 303 of 308 brain regions, which belonged to Yeo 7 functional networks including the somatomotor, default mode, limbic, frontoparietal, ventral attention, dorsal attention, and visual network, were matched and then included in the following procedures. Please refer to Table S3 for information on those unmatched and discarded regions.

For each brain region, 9 structural features, i.e., fractional anisotropy, mean curvature, Gaussian curvature, cortical thickness, surface area, gray matter volume, mean diffusivity, folding index, and curved index, were calculated from the T1 and DTI images, respectively. For resting-state functional images, the regional homogeneity, the fractional amplitude of low-frequency fluctuations, and the amplitude of low-frequency fluctuation in each region were computed by averaging the values of all voxels in the region. As for task fMRI images, as well as resting and task EEG data, the time series per region were extracted and the mean time series of all voxels in this region were calculated. To determine functional interactions, for the fMRI time series, Pearson’s correlation between paired regions of interest (ROIs) was computed, while the phase-locking value was used to evaluate the interregional phase synchronization for source EEG signals. Thereafter, based on the networks of both fMRI and EEG, multiple nodal parameters, including eigenvector centrality, closeness centrality, betweenness centrality, and degree centrality, were calculated via the brain connectivity toolbox (BCT; http://www.nitrc.org/projects/bct/) [71].

Afterward, each functional and morphometric feature vector was *z*-normalized, and Pearson’s correlations were applied to the merged functional and morphometric feature vector for paired ROIs, leading to 303 × 303 adjacent matrices for each participant. Hence, for 303 ROIs of the D-K atlas, the corresponding *MCN* ∈ R^303×303^ can be calculated as follows:

$$\mathrm{MCN}=\left[\begin{array}{cccc} w_{1,1}^{\mathrm{PCC}} & w_{1,2}^{\mathrm{PCC}} & \cdots & w_{1,303}^{\mathrm{PCC}}\\ w_{2,1}^{\mathrm{PCC}} & w_{2,2}^{\mathrm{PCC}} & \cdots & w_{2,303}^{\mathrm{PCC}}\\ \vdots & \vdots & \cdots & \vdots \\ w_{303,1}^{\mathrm{PCC}} & w_{303,2}^{\mathrm{PCC}} & \cdots & w_{303,303}^{\mathrm{PCC}} \end{array}\right]$$ (1)

where $w_{i,j}^{\mathrm{PCC}}$is Pearson’s correlation coefficient between *roi_i_* and *roi_j_*.

Based on the constructed MCNs, network measures were calculated as well. Here, an adapted community detection algorithm [72] with an additional “final-tuning” algorithm [73] was applied to estimate the average network modularity across 100 runs of this algorithm. Then, network properties, i.e., *C*, *Le*, *Ge*, and *L*, which evaluate the potential for functional segregation and integration, were estimated by the BCT [71]. Furthermore, to validate the repeatability of MCN construction, resting-state MCNs of HCs in these 2 independent cohorts (i.e., the discovery and validation cohort) were statistically correlated with each other by using Person’s correlation.

### Group comparisons of MCNs

Based on the MCN adjacency matrix, the sum of the weighted linkages between a given region and its connections to others was calculated as the regional MCN. To mine the potential differences of MCN under different brain states (i.e., resting versus task) for the discovery cohort, paired-sample *t* tests were performed with an false discovery rate (FDR) correction, and *t* map (contrast = task − rest) was generated.

For the validation cohort, owing to some technical variability and systematic bias attributed to different scanners and imaging parameters, the multimodal parameters, such as DTI fractional anisotropy and MRI cortical thickness, could differ among the 4 sites (i.e., UM, TX, CU, and MG). Therefore, the ComBat method [74,75] was applied to the 4 imaging datasets. Notably, to further control the confounding of the site with age and gender, we first regressed out the variation explained by age and gender. As such, regional MCNs were combined from 4 sites for MDD patients and healthy individuals, respectively, and based on these individual MCNs, the independent sample *t* tests with an FDR correction were conducted to capture any potential MCN differences between MDDs and HCs.

### Classification of MDDs and HCs

The SPN approach [26] was first applied to extract the discriminative features from MCNs of MDD patients and HCs. Thereafter, the corresponding SPN features were used to train the linear discriminant analysis (LDA) classifier that would be applied in the individual-level classification of patients with MDD and healthy populations. To evaluate the classification performance, following the protocols reported previously [76], the leave-one-out cross-validation (LOOCV) strategy was used.

### Transcriptomic and genomic analyses

The AHBA transcriptomic dataset (http://human.brain-map.org) [77] was used in this study. The corresponding preprocessing was performed according to a recommended standard protocol (https://github.com/BMHLab/AHBAprocessing) [16]. Meanwhile, given that only 2 right hemispherical data were included in the AHBA dataset, following the previous studies [11,15], only the left hemisphere was considered. Thereafter, to determine the relationship between transcriptional levels for all 10,027 genes and the regional MCN changes, PLS regression was adopted, and the expression levels of 10,027 genes were applied as predictor variables to predict the regional MCN alterations. The first PLS component (PLS1) represented the cortical expression map that is strongest related to the MCN differences. Additionally, permutation testing was implemented to calculate the statistical significance of the variance explained by the PLS component (5,000 times) [20]. To assess the contribution of each gene to PLS1, the bootstrapping procedure was adopted. The ratio of the weight of each gene to its bootstrap standard error was applied to compute the *Z* score for each gene, and the genes were then ranked by their contributions to PLS1 [20]. Two sets of candidate genes with an FDR < 0.05 were generated, corresponding to the regional MCN changes either positively, PLS1^+^ (*Z* > 5), or negatively, PLS1^−^ (*Z* < −5). In addition, Metascape analysis was performed to estimate the enrichment of KEGG pathways and GO biological processes [24] for the PLS1^+^ and PLS1^−^ genes, respectively. The enrichment pathways were considered under a significance level of 0.01 and further corrected by FDR.

The significant PLS1^+^/PLS1^−^ genes generated from the discovery and validation cohorts were also compared with the candidate gene lists of a large-scale meta-analysis of education attainment GWAS [25] and 3 combined MDD GWAS studies [27–29]. A permutation test [78] was first applied to estimate the *P* values of overlapped genes between the PLS1^+^/PLS1^−^ genes and the genes related to human cognition or MDD from the GWAS results. Then, a multi-gene list meta-analysis [24] was applied to test whether the PLS1^+^ (PLS1^−^) genes shared enrichment pathways with the GWAS-derived results, which were thresholded at 0.01 with FDR correction.

### Genes expressed in specific cell types

Given the cellular diversity of the human brain, an indirect method [19] was performed in the current work to allocate the PLS1^+^ (PLS1^−^) gene list to 7 canonical cell classes. Specifically, data from 5 different single-cell studies were compiled to acquire genes of each cell type, and following Seidlitz et al. [19], we reorganized cell types into 7 canonical classes. Then, we overlapped the PLS1^+^ (PLS1^−^) gene list with genes of each cell type, respectively, to assign cognition- or MDD-specific gene lists to cell types. To calculate the *P* value of the number of overlapped genes in each cell type, permutation tests [78] were performed with FDR correction. Subsequently, enrichment analysis was applied to investigate the enrichment pathways in genes involved in each cell type, which were thresholded at 0.01 and corrected by the FDR.

### Validation analysis

Following the identical strategy used for the D-K atlas, S-MCN, E-MCN, SF-MCN, SE-MCN, FE-MCN, and SFE-MCN were also constructed based on the human Brainnetome atlas [31]. The network modularity and properties were quantitatively calculated and statistically compared by the paired-sample *t* tests. Then, the task-rest *t* map was obtained and PLS regression was adopted to map these MCN variations to anatomically patterned gene expression from the AHBA. To further verify the gene lists obtained by MCN analysis, a multi-gene list meta-analysis [24] was conducted between the PLS1^+^ (PLS1^−^) gene lists obtained from the D-K and Brainnetome atlas. All obtained pathways were thresholded for significance at 5%, corrected by the FDR.

## Data Availability

The transcriptomic dataset can be found at: https://human.brainmap.org/static/download. The MRI-EEG data of the validation cohort (EMBARC data) are publicly available at https://nda.nih.gov/edit_collection.html?id=2199. Depression-related genes are available at http://help.brain-map.org/download/attachments/2818165/HBA_ISH_GeneList.pdf?version=1&modificatioDate=1348783035873&api=v2. Compiled cell-specific gene set list is available at https://staticcontent.springer.com/esm/art%3A10.1038%2Fs41467-020-170515/MediaObjects/4 1467_2020_17051_MOESM8_ESM.xlsx. Supplementary Data 1 provided the PLS1 genes of the discovery cohort. Other information is available upon reasonable request.

## Supplementary Materials

Supplementary 1 Supplementary Methods Supplementary Results Tables S1 to S9 Figs. S1 to S11
