# Supplementary material for: Transcriptomic and Macroscopic Architectures of Multimodal Covariance Network Reveal Molecular–Structural–Functional Co-alterations
Source: Research (Wash D C). 2023 Jun 8;6:0171. doi: 10.34133/research.0171 (PMC10249784; doi:10.34133/research.0171)
Supplement: Supplementary 1 — Supplementary Methods Supplementary Results Tables S1 to S9 Figs. S1 to S11 [file research.0171.f1.docx]

Supplementary Materials

**Supplementary Methods**

*1. Detailed MRI parameters for the discovery cohort*

**Table S1.** MRI parameters for the discovery cohort

| Structural | Diffusion | Functional |
| --- | --- | --- |
| General Electric 3T | General Electric 3T | General Electric 3T |
| TR = 6.0 ms | TR = 8500 ms | TR = 2000 ms |
| TE = 2.0 ms | TE = 64.1 ms | TE = 30 ms |
| Flip angle = 9° | Flip angle = 90° | Flip angle = 90° |
| FOV = 205 × 205 mm | FOV = 256 × 256 mm | FOV = 240 × 240 mm |
| Slice thickness = 1 mm | Matrix = 128 × 128 | Slice thickness = 4 mm |
| Matrix = 256 × 256 | voxel size = 2.0 × 2.0 × 2.0 mm | Matrix = 64 × 64 |
| voxel size = 1.0 × 1.0 × 1.0 mm | 67 slices | voxel size = 3.75 × 3.75 × 4 mm |
| 154 slices | *b* value = 1000 s/mm^2^ | 35 slices |

*2. Detailed MRI parameters for the four clinical sites of the validation cohort*

**Table S2.** MRI parameters for the four clinical sites

| Scanner | CU | MG | TX | UM |
| --- | --- | --- | --- | --- |
|  | General Electric 3T | Siemens 3T | Phillips 3T | Phillips 3T |
| Structural | FSPGR | MPRAGE | Turbo field echo | MPRAGE |
|  | TR = 6.0 ms | TR = 2300 ms | TR = 8.2 ms | TR = 2100 ms |
|  | TE = 2.4 ms | TE = 2.54 ms | TE = 3.7 ms | TE = 3.7 ms |
|  | TI = 900 ms | TI = 900 ms | TI = 1100 ms | TI = 1100 ms |
|  | Flip angle = 9° | Flip angle = 9° | Flip angle = 12° | Flip angle = 12° |
|  | FOV = 256 × 256 mm | FOV = 256 × 256 mm | FOV = 256 × 256 mm | FOV = 256 × 256 mm |
|  | Slice thickness = 1 mm | Slice thickness = 1 mm | Slice thickness = 1 mm | Slice thickness = 1 mm |
|  | Matrix = 256 × 256 | Matrix = 256 × 256 | Matrix = 256 × 256 | Matrix = 256 × 256 |
|  | 174 continuous slices | 176 continuous slices | 178 continuous slices | 178 continuous slices |
| Functional | TR = 2000 ms | TR = 2000 ms | TR = 2000 ms | TR = 2000 ms |
|  | TE = 28 ms | TE = 28 ms | TE = 28 ms | TE = 28 ms |
|  | Flip angle = 90° | Flip angle = 90° | Flip angle = 90° | Flip angle = 90° |
|  | FOV = 205 × 205 mm | FOV = 205 × 205 mm | FOV = 205 × 205 mm | FOV = 205 × 205 mm |
|  | Slice thickness = 3.1 mm | Slice thickness = 3.1 mm | Slice thickness = 3.1 mm | Slice thickness = 3.1 mm |
|  | Matrix = 64 × 64 | Matrix = 64 × 64 | Matrix = 64 × 64 | Matrix = 64 × 64 |
| Diffusion | TR = 13100 ms | TR = 8310 ms | TR = 9500 ms | TR = 9500 ms |
|  | TE = 75.6 ms | TE = 95 ms | TE = 95 ms | TE = 96 ms |
|  | Flip angle = 90° | Flip angle = 90° | Flip angle = 90° | Flip angle = 90° |
|  | Slice thickness = 2.5 mm | Slice thickness = 2.5 | Slice thickness = 2.5 | Slice thickness = 2.5 |
|  | Matrix = 96 × 96 | Matrix = 96 × 96 | Matrix = 96 × 96 | Matrix = 96 × 96 |
|  | *b* value = 1000 s/mm^2^ | *b* value = 1000 s/mm^2^ | *b* value = 1000 s/mm^2^ | *b* value = 1000 s/mm^2^ |

*3. The unmatched and discarded regions of the Desikan-Killiany atlas*

**Table S3.** The unmatched and discarded regions of the Desikan-Killiany atlas

| ROI | MNI | | | Structural name |
| --- | --- | --- | --- | --- |
|  | X | Y | Z |  |
| 1 | -39.72 | 11.34 | 48.85 | lh_caudalmiddlefrontal_part4 |
| 1 | -48.10 | 30.82 | 5.23 | lh_parstriangularis_part1 |
| 3 | -40.49 | 31.11 | -0.50 | lh_parstriangularis_part2 |
| 4 | -35.45 | 4.28 | -0.59 | lh_insula_part4 |
| 5 | 23.85 | -65.16 | 38.59 | rh_superiorparietal_part5 |

**Supplementary Results**

*1. Correlations between the MCN properties and task performance/clinical scales.* We first explored the relationship between the task multimodal covariance network (MCN) properties (i.e., the clustering coefficient (*C*), characteristic path length (*L*), global efficiency (*Ge*), and local efficiency (*Le*)) and the task behaviors (i.e., the risky rate) during the gambling task for the discovery cohort. As listed in Table S4, although the SE-MCN, FE-MCN, and SFE-MCN properties were all significantly related to the risky performance (*p* < 0.05), the network properties of the SFE-MCN exhibited the highest correlation coefficients with the risky rates. Concerning other MCNs, i.e., the S-MCN, E-MCN, and SF-MCN, no such correlations between the network properties and risky rates could be identified (*p* > 0.05).

**Table S4.** Correlations between the task MCN properties and risky rates in the discovery cohort

|  |  | S-MCN | E-MCN | SF-MCN | SE-MCN | FE-MCN | SFE-MCN |
| --- | --- | --- | --- | --- | --- | --- | --- |
| *C* | *r* | 0.34 | 0.36 | 0.08 | 0.40 | 0.41 | 0.51 |
|  | *p* | 0.10 | 0.07 | 0.71 | 0.05 | 0.04 | 0.01 |
| *L* | *r* | -0.30 | -0.20 | -0.06 | -0.47 | -0.48 | -0.53 |
|  | *p* | 0.15 | 0.35 | 0.76 | 0.02 | 0.02 | 0.01 |
| *Ge* | *r* | 0.32 | 0.31 | 0.13 | 0.39 | 0.44 | 0.54 |
|  | *p* | 0.12 | 0.13 | 0.54 | 0.05 | 0.03 | 0.004 |
| *Le* | *r* | 0.33 | 0.35 | 0.09 | 0.40 | 0.41 | 0.52 |
|  | *p* | 0.10 | 0.09 | 0.68 | 0.05 | 0.04 | 0.01 |

As for major depression disorder (MDD) patients in the validation cohort, only the SFE-MCN properties were found to be significantly correlated with the Hamilton Depression Rating Scale (HAMD; *p* < 0.05), no such correlations between the HAMD scores and properties of the S-MCN, E-MCN, SF-MCN, SE-MCN, or FE-MCN were found (*p* > 0.05; Table S5).

**Table S5.** Correlations between the MCN properties of the MDD and HAMD scores in the validation cohort

|  |  | S-MCN | E-MCN | SF-MCN | SE-MCN | FE-MCN | SFE-MCN |
| --- | --- | --- | --- | --- | --- | --- | --- |
| *C* | *r* | -0.03 | -0.03 | 0.05 | 0.12 | 0.14 | -0.18 |
|  | *p* | 0.73 | 0.72 | 0.54 | 0.11 | 0.06 | 0.01 |
| *L* | *r* | 0.08 | 0.01 | 0.001 | -0.06 | -0.12 | 0.18 |
|  | *p* | 0.31 | 0.91 | 0.97 | 0.39 | 0.12 | 0.01 |
| *Ge* | *r* | -0.04 | -0.02 | 0.04 | 0.12 | 0.15 | -0.21 |
|  | *p* | 0.63 | 0.74 | 0.60 | 0.10 | 0.05 | 0.004 |
| *Le* | *r* | -0.03 | -0.03 | 0.05 | 0.12 | 0.14 | -0.19 |
|  | *p* | 0.74 | 0.73 | 0.56 | 0.10 | 0.05 | 0.01 |

*2. Spatial similarity of regional MCN between the discovery and validation cohort.* To explore the replicability of regional MCN, the spatial similarity of the grand-averaged regional MCN between the two independent cohorts was further calculated by Pearson’s correlation. The corresponding Pearson’s correlation coefficients *r* and *p* values are statistically calculated and listed in Table S6, and the spatial autocorrelation was further corrected by a “*spin*”-based method [23]. We found that with the addition of more modalities, the replicability of MCNs between the two cohorts consistently increased. Particularly, among these MCNs, the spatial patterns of the regional SFE-MCN showed the strongest correlation between the two groups of healthy participants (*r*_(301)_ = 0.96, *p_spin_* < 0.001), suggesting the promoted replicability of SFE-MCN in detecting the structural-functional covarying of the human brain.

**Table S6.** Spatial correlations of the regional MCN between the discovery and validation cohort

|  | S-MCN | E-MCN | SF-MCN | SE-MCN | FE-MCN | SFE-MCN |
| --- | --- | --- | --- | --- | --- | --- |
| *r* | 0.81 | 0.76 | 0.93 | 0.83 | 0.89 | 0.96 |
| *p_spin_* | *p_spin_* < 0.001 | *p_spin_* < 0.001 | *p_spin_* < 0.001 | *p_spin_* < 0.001 | *p_spin_* < 0.001 | *p_spin_* < 0.001 |

*3. Symmetry of the regional MCN alterations.* Herein, to explore the symmetry of the regional MCN alterations between the left and right hemispheres, we first statistically probed the inter-hemispherical differences in the regional MCN alterations (i.e., the task-rest *t*-values of the discovery cohort and the case-control *t*-value of the validation cohort) by performing a paired-sample *t*-test. Results show that, for either the discovery or validation cohort, the *t*-values were significantly different between the two hemispheres (discovery cohort: *p* < 0.01; validation cohort: *p* < 0.01), suggesting the asymmetric MCN patterns of the human brain. In addition, we further explored the relationship of the regional MCN alterations between the left and right hemispheres by Pearson’s correlation. As provided in Figure S1, we found a moderate correlation between the left and right *t*-values (discovery cohort: *r* = 0.43; *p* < 0.001; validation cohort: *r* = 0.52; *p* < 0.001).


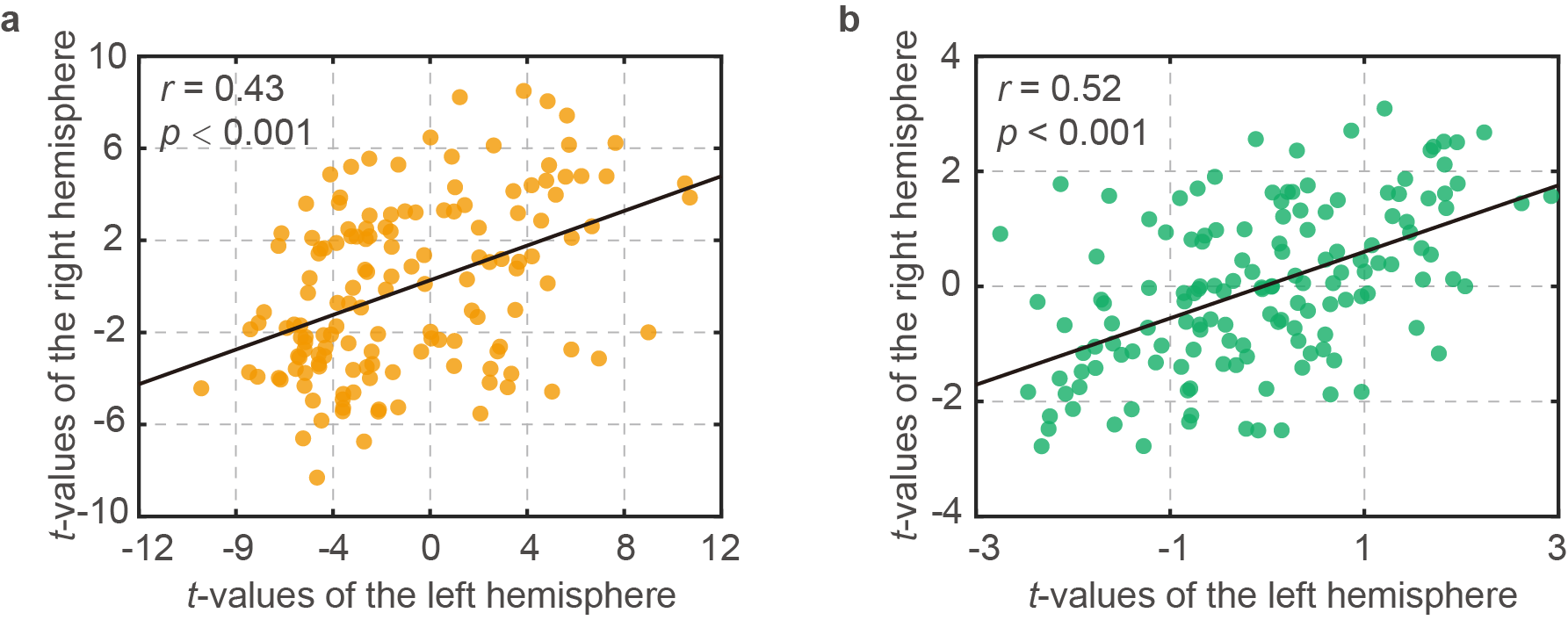


**Figure S1.** The symmetry of the regional MCN alterations between the left and right hemispheres. **a**. Correlation of the task-rest *t*-values between the left and right hemispheres in the discovery cohort. **b**. Correlation of the case-control *t*-values between the left and right hemispheres in the validation cohort. The black line represents the fitted curve, and the colored circles denote the participants.

*4. Statistical significance of the variance explained by the PLS component.* In this work, the PLS regression was adopted to determine the relationship between transcriptional levels for all 10,027 genes and the regional MCN changes. Thereinto, the statistical significance of the variance explained by each PLS component was calculated by permuting the response variables 5000 times and the *p*-value was accordingly reported (*p_perm_*) [20]. Results show that the first PLS component explained 32% of the variance in the task-rest regional MCN differences of the discovery cohort (*p_perm_* < 0.001; Table S7), and PLS1 explained 34% of the variance in the case-control regional MCN differences for the validation cohort (*p_perm_* < 0.001; Table S8), significantly exceeding the expected by chance. However, for both cohorts, no statistical significance was achieved for the PLS2 and PLS3 components (*p_perm_* > 0.05).

**Table S7.** The explainability and statistical significance of the first three PLS components in the discovery cohort

|  | PLS1 | PLS2 | PLS3 |
| --- | --- | --- | --- |
| *Variance explained by PLS component* | 32% | 12% | 6% |
| *p_perm_* | *p_perm_* < 0.001 | *p_perm_* = 0.20 | *p_perm_* = 0.96 |

**Table S8.** The explainability and statistical significance of the first three PLS components in the validation cohort

|  | PLS1 | PLS2 | PLS3 |
| --- | --- | --- | --- |
| *Variance explained by PLS component* | 34% | 15% | 13% |
| *p_perm_* | *p_perm_* < 0.001 | *p_perm_* = 0.06 | *p_perm_* = 0.29 |

*5. Enrichment analysis of PLS1- genes in the discovery cohort.* After discarding discrete enrichment clusters and correcting for enrichment terms (*p_FDR_* < 0.01), the enrichment results of PLS1- genes were exhibited in Figure S2. Concretely, these negatively weighted gene sets were enriched for five GO biological processes including “regulation of transmembrane transport”, “inorganic ion transmembrane transport”, and “action potential”, as well as three KEGG pathways, such as the “MAPK signaling pathway”.


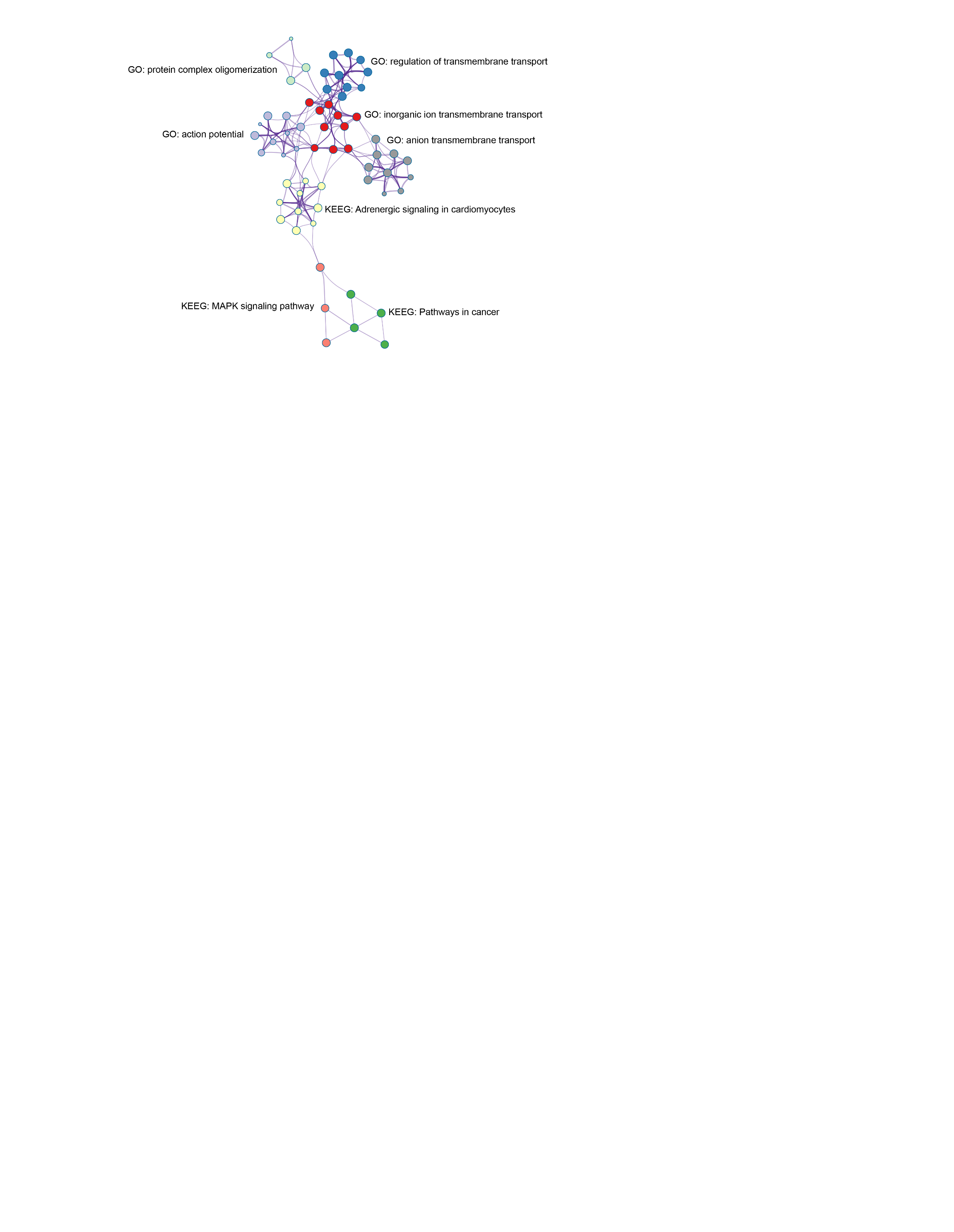


**Figure S2.** Enrichment pathways of negatively weighted genes with Z < -5.

*6. Multi-gene-list meta-analysis between cognitive task-related genes and that from GWAS.* In this work, we validated whether there are shared enrichment pathways between the PLS1+ (PLS1−) gene list and genes that were significantly related to educational attainment and cognitive performance [25]. Specifically, by the multi-gene-list meta-analysis [24], we observed that enrichment pathways of the PLS1+ genes consisted of 17 of 20 pathways of genes from GWAS studies, while that of the PLS1- gene list consisted of 15 of 20 pathways. The overlapped distribution is exhibited in Figure S3b and S4b, respectively. The enrichment pathways included “synaptic signaling”, “brain development”, “regulation of ion transport”, and “behavior”, “regulation of neuron projection development”, and “cell junction organization” (Figure S3a and S4a).


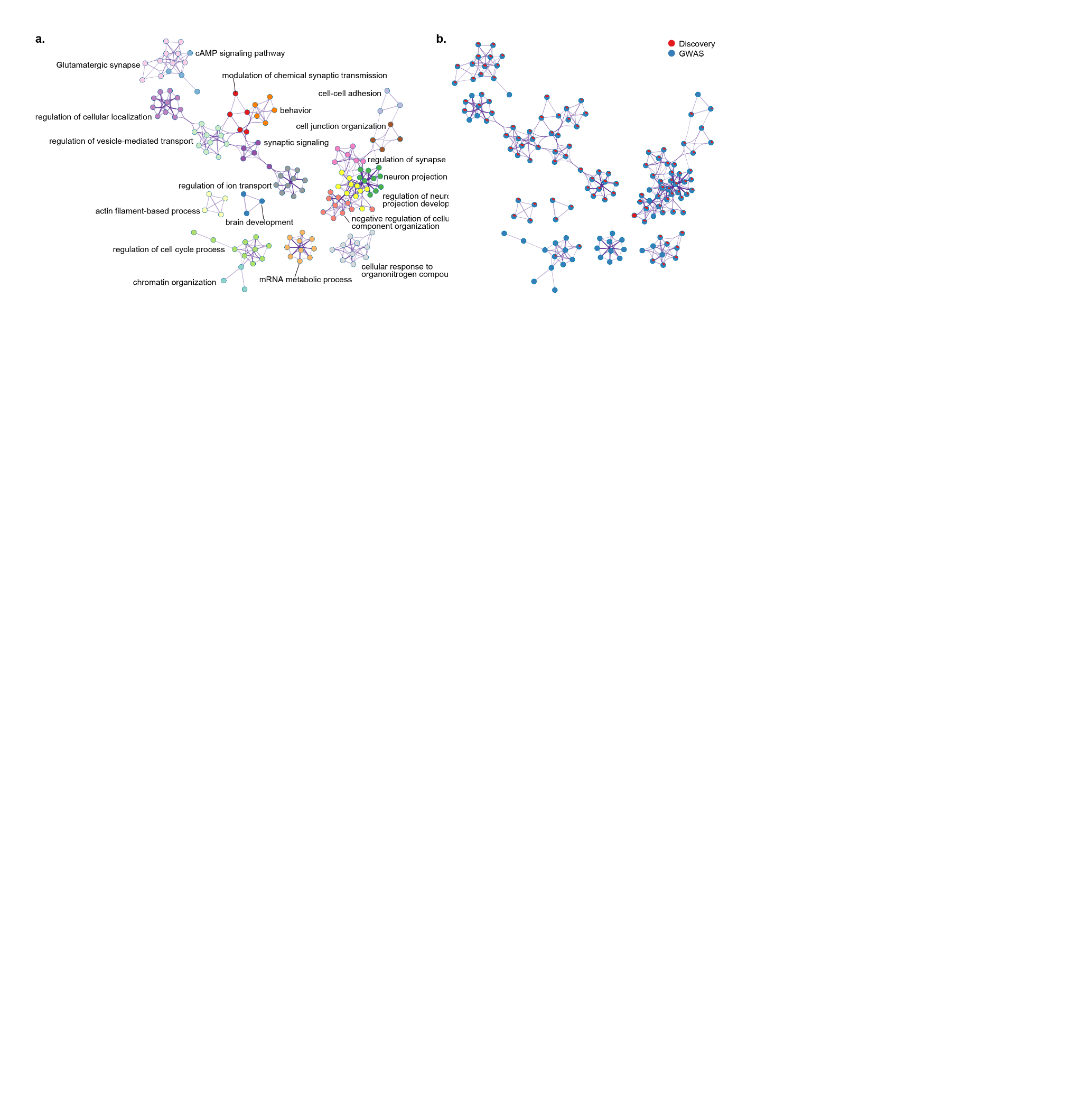


**Figure S3.** Overlapped ontology terms between the PLS1+ gene list of the discovery cohort and GWAS studies. **a.** The subset of ontology terms. **b.** The overlapped distribution.


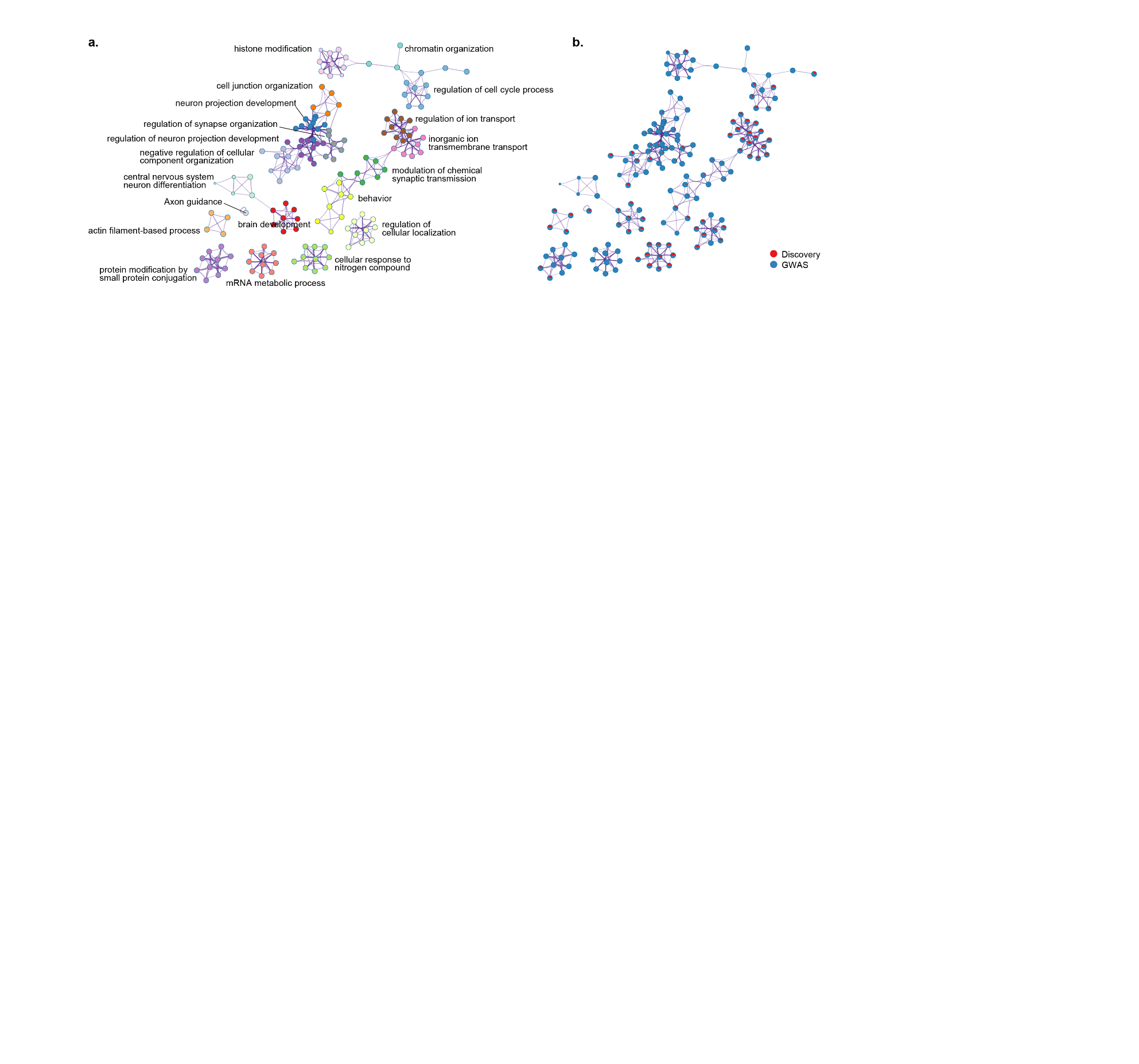


**Figure S4.** Overlapped ontology terms between the PLS1- gene list of the discovery cohort and GWAS studies. **a.** The subset of ontology terms. **b.** The overlapped distribution.

*7. Enrichment analysis of PLS1- genes in the validation cohort.* For the PLS1- gene list (*Z* < - 5) in the validation cohort, after discarding discrete enrichment clusters and correcting for enrichment terms (*p_FDR_* < 0.01), the enrichment results of PLS1- genes were presented in Figure S5. Concretely, the PLS1- gene list was enriched for 7 GO biological processes including the “regulation of system process”, “regulation of membrane potential”, and “trans-synaptic signaling”, while no KEGG pathways were enriched for the PLS1- gene list.


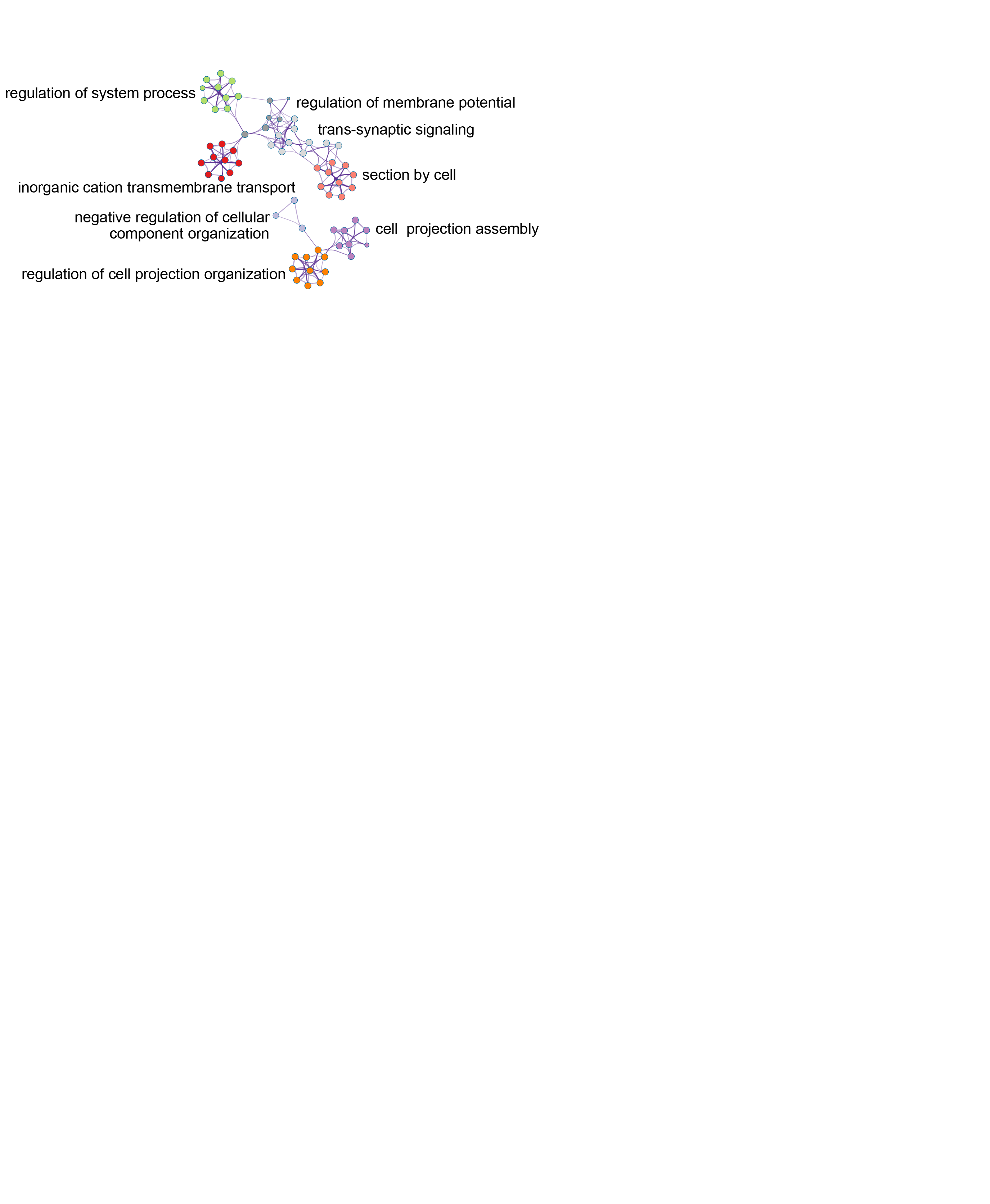


**Figure S5.** Enrichment pathways of negatively weighted genes with Z < -5.

*8. Multi-gene-list meta-analysis between MDD-related genes and that from GWAS.* Likewise, data from three recent GWAS studies with genes relating to the MDD phenotype [27-29] were compiled. By the multi-gene-list meta-analysis, we observed that enrichment pathways of the PLS1+ (PLS1-) gene list consisted of 13 of 19 pathways of genes from GWAS studies. The overlapped distribution is exhibited in Figure S6 and S7, respectively, which included “synaptic signaling”, “head development”, “regulation of ion transport”, “cell-cell adhesion”, “regulation of cycle process”, and “cell junction organization”.


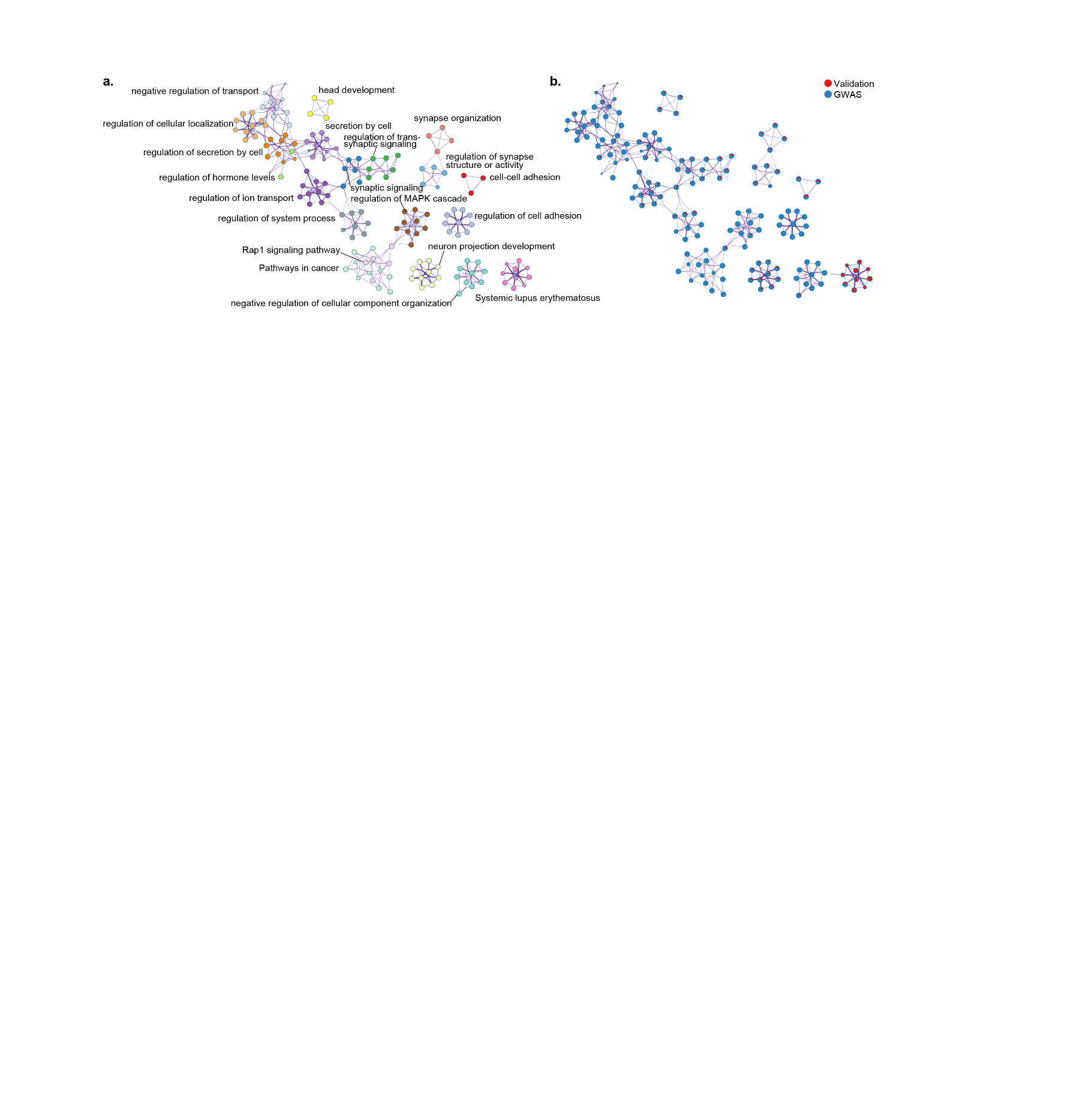


**Figure S6.** Overlapped ontology terms between the PLS1+ gene ist of the validation cohort and GWAS studies. **a.** The subset of ontology terms. **b.** The overlapped distribution.


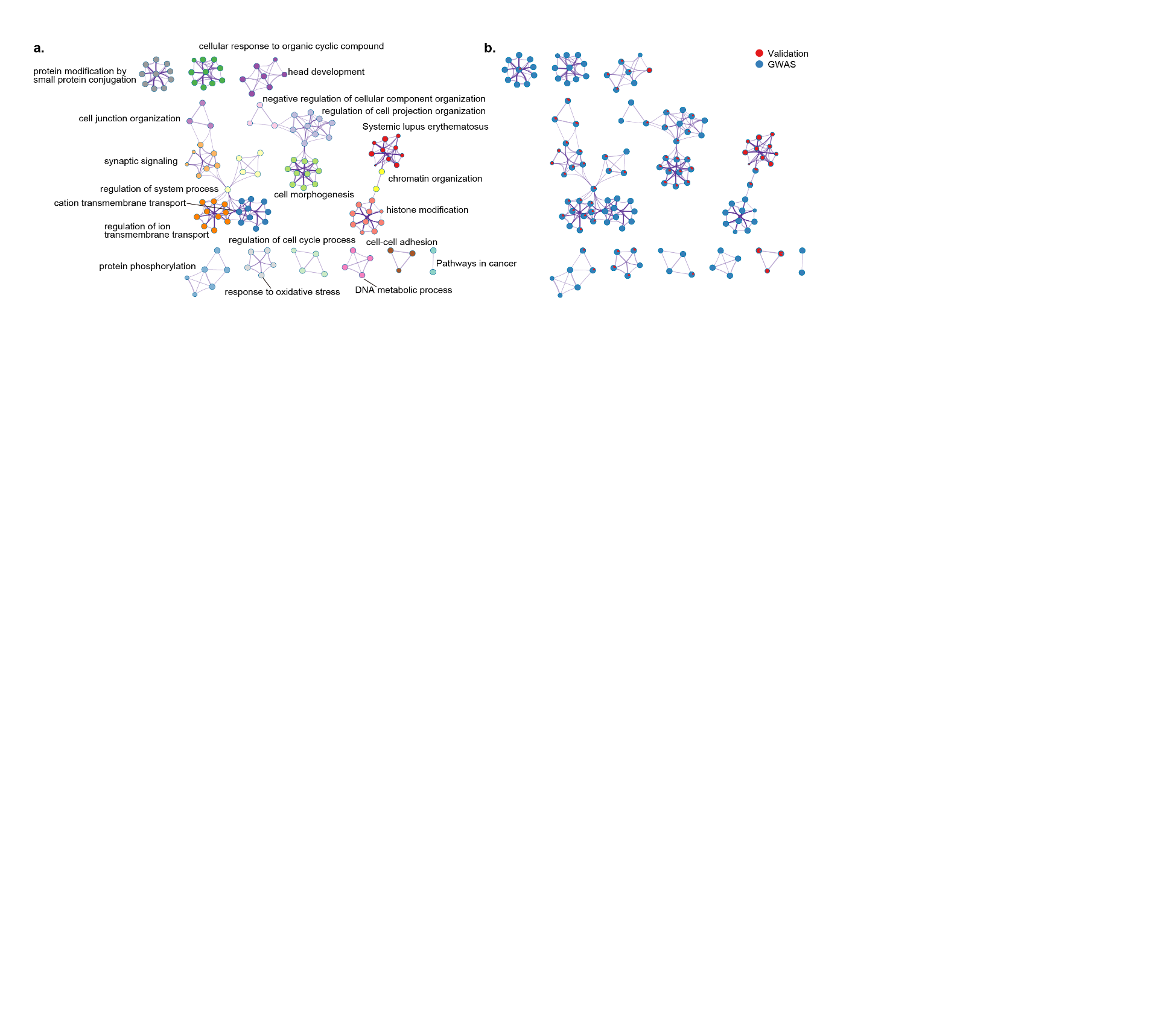


**Figure S7.** Overlapped ontology terms between the PLS1- gene ist of the validation cohort and GWAS studies. **a.** The subset of ontology terms. **b.** The overlapped distribution.

*9. Relationships between MDD-related MCN alterations and genes from in situ hybridization in the adult human brain.* In this work, 24 MDD-related genes were first identified from in situ hybridization data in the AHBA (help.brain-map.org/display/humanbrain/Documentation) [30]. Among these genes, 12 genes were found within a total of 10,027 genes, and 10 out of the 12 known MDD genes showed significant correlations with regional changes in MCN (*p_FDR_* < 0.05; Figure S8), including four negative correlations (i.e., *CUX2, CHRM2, HTR5A, and ARRA2A*) and six positive correlations (i.e., *HTR1A, CNR1, SST, TAC1, PDE1A, and MAOA*).


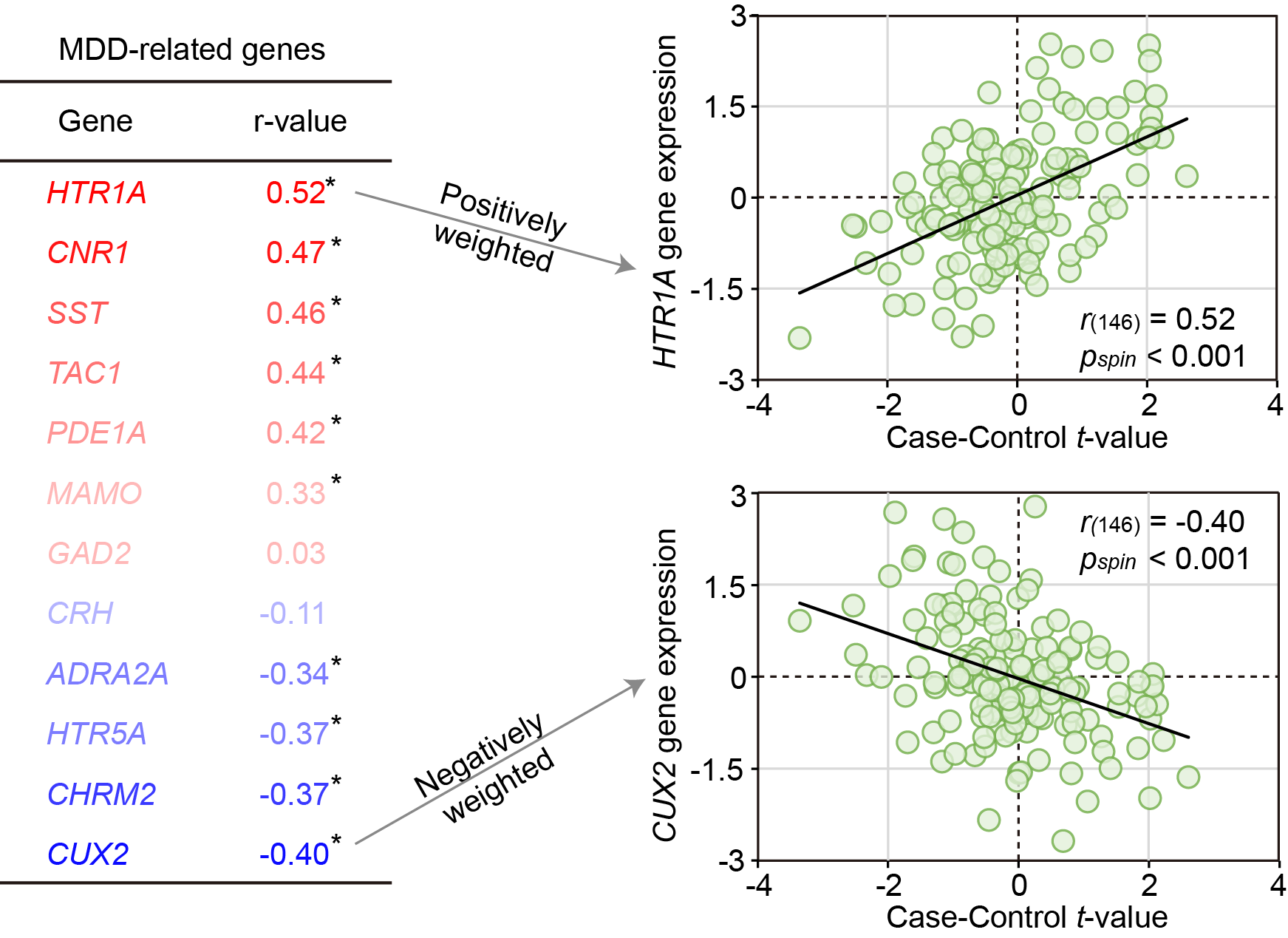


**Figure S8.** MDD-related genes from in situ hybridization in the adult human brain positively and negatively correlated with regional changes in MCN. *, *p* < 0.05, FDR-corrected.

*10. Cellular characterization of the depression-specific genes.* Afterward, we found that genes in the PLS1+ gene list were expressed primarily in astrocytes (156 genes, *p_perm_* < 0.01) and microglia (113 genes, *p_perm_* < 0.01) (Figure S9a), respectively; while genes specific to excitatory neurons (228 genes, *p_perm_* < 0.01) and inhibitory neurons (161 genes, *p_perm_* < 0.01) were overrepresented in the PLS1– gene set (Figure S9b). Consistently, Metascape analysis of cell-type-specific genes uncovered that changes in MDD-MCN were significantly enriched for synapse function and neuroinflammation-related processes in astrocytes, microglia, and neurons, such as “synapse organization”, “signaling pathway”, and “synaptic signaling”.


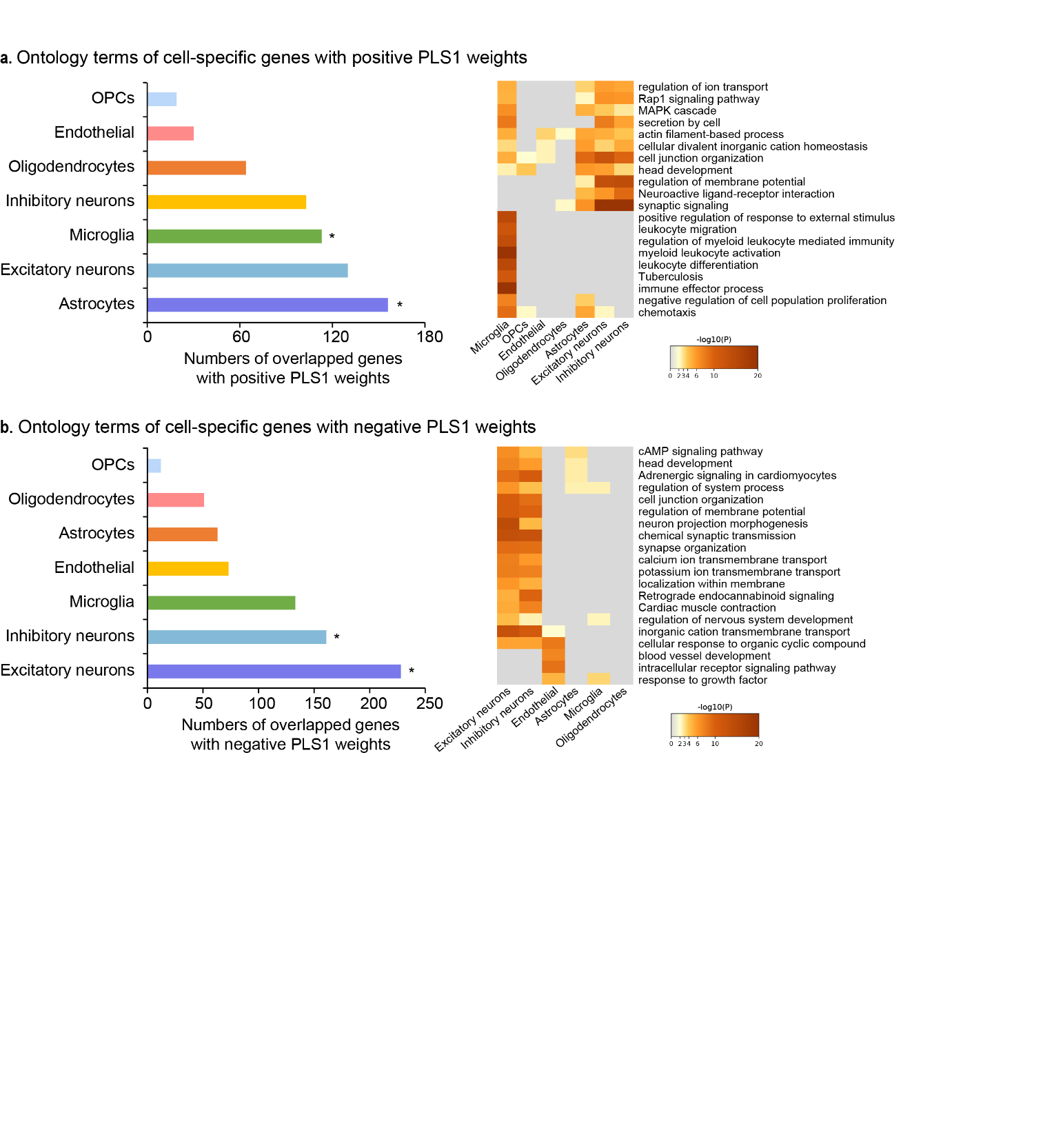


**Figure S9.** Cell type specificity of MDD-related MCN alterations. Enrichment analysis of cell-specific genes with PLS1 + (**a**) and PLS1− (**b**) weights. The left panel represents the number of overlapping genes in each cell type. *, *P_perm_* < 0.05, FDR-corrected. The right panel represents the enrichment pathways for each cell type.

*11. Reproducibility of SFE-MCN superiority and corresponding transcriptomic profile*. For validation, the human Brainnetome atlas [31] with 210 cortical subregions, which provides a fine-grained, cross-validated atlas and contains information on both structure and function, was further applied to validate the repeatability of the above results on the discovery cohort. Specifically, following the identical procedures used for the D-K atlas, multiple functional and morphometric features were extracted for the Brainnetome atlas, and the S-MCN, E-MCN, SF-MCN, SE-MCN, FE-MCN, and SFE-MCN were accordingly constructed. As exhibited in Figure S10a, the spatial patterns of MCN constructed by the Brainnetome atlas were similar to those constructed by the D-K atlas, particularly that, the network modularity was consistently increased with the fusion of more brain modalities (*p* < 0.05; Figure S10b). Subsequently, significantly larger modular parameters of the resting state in contrast to the task state were found for both the SF-MCNs and SFE-MCNs (*p* < 0.05; Figure S10c), whose properties were further significantly related to the risky rates of the gambling task (*p* < 0.05; Table S9 and Figure S10d). Namely, these results consistently validate the superiority and repeatability of SFE-MCN in capturing the cognition-specific structural-functional covarying of the human brain by using a different brain atlas, further providing high sensitivity in reflecting individual cognitive performance.


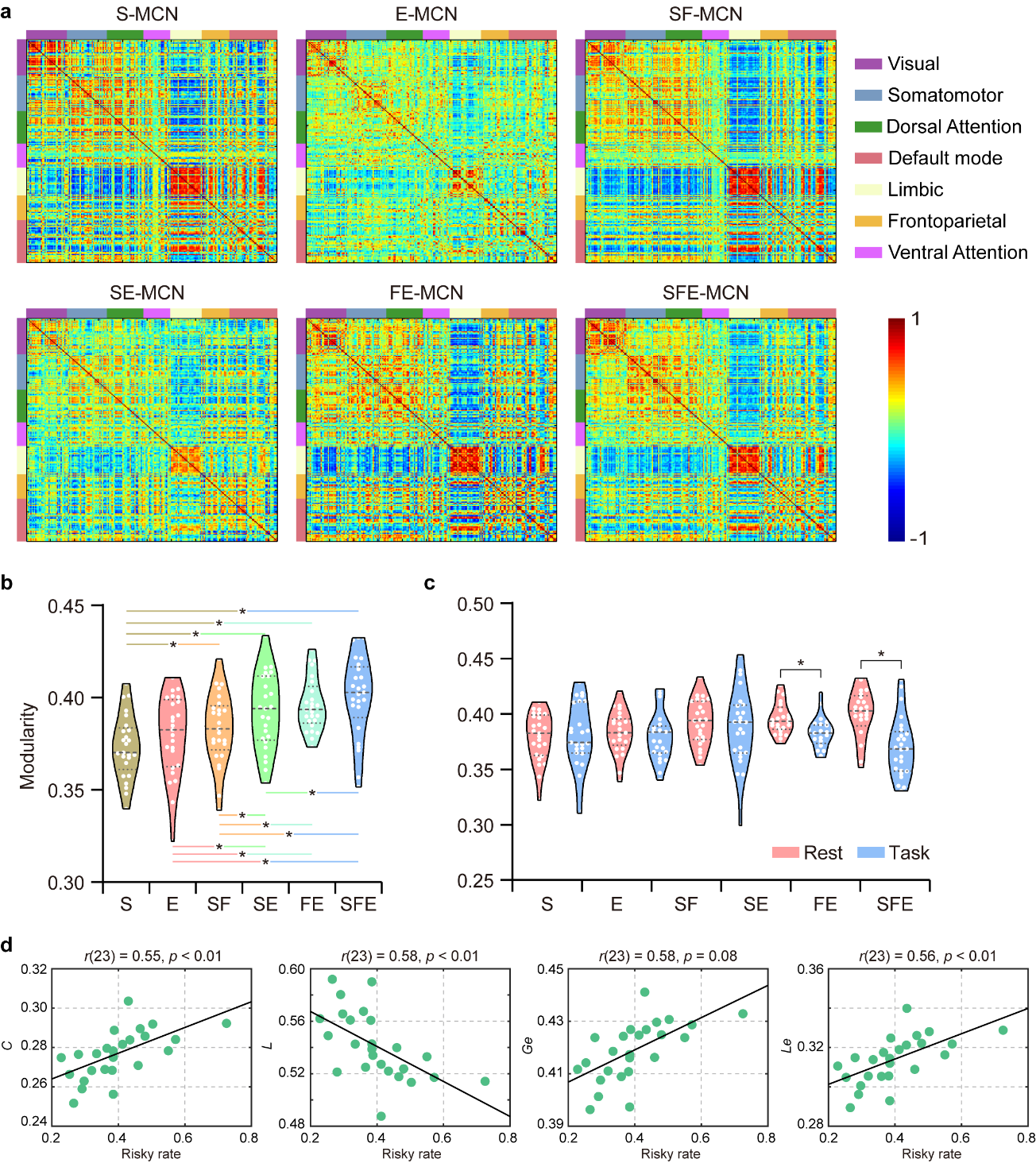


**Figure S10.** Multimodal covariance network (MCN) constructed by the human Brainnetome atlas for the discovery cohort. **a.** Resting-state MCNs constructed by the human Brainnetome atlas. S-MCN: MCN constructed by sMRI; E-MCN: MCN constructed by EEG; SF-MCN: MCN constructed by sMRI and fMRI; SE-MCN: MCN constructed by sMRI and EEG; FE-MCN: MCN constructed by fMRI and EEG; SFE-MCN: MCN constructed by sMRI, fMRI, and EEG. **b.** The modularity of various MCNs. The colored violins represent the modularity of the S-MCN, E-MCN, SF-MCN, SE-MCN, FE-MCN, and SFE-MCN, respectively, and the binary color-coded line with asterisk reflects a significant modularity difference between two corresponding MCNs represented by the two colors (*p* < 0.05). **c.** The MCN modularity of the resting state and gambling task. The asterisk indicates the significant difference in the modularity (*p* < 0.05). **d.** Relationships between the task SFE-MCN properties and risky rates. The black line represents the fitted curve, and the colored circles denote the participants.

**Table S9.** Correlations between the task MCN properties and risky rates in the discovery cohort

|  |  | S-MCN | E-MCN | SF-MCN | SE-MCN | FE-MCN | SFE-MCN |
| --- | --- | --- | --- | --- | --- | --- | --- |
| *C* | *r* | 0.25 | 0.28 | 0.16 | 0.29 | 0.44 | 0.55 |
|  | *p* | 0.22 | 0.18 | 0.43 | 0.16 | 0.03 | 0.004 |
| *L* | *r* | -0.23 | -0.09 | -0.15 | -0.29 | -0.49 | -0.58 |
|  | *p* | 0.26 | 0.69 | 0.46 | 0.17 | 0.01 | 0.002 |
| *Ge* | *r* | 0.22 | 0.17 | 0.21 | 0.31 | 0.47 | 0.58 |
|  | *p* | 0.29 | 0.41 | 0.31 | 0.13 | 0.02 | 0.003 |
| *Le* | *r* | 0.34 | 0.25 | 0.17 | 0.34 | 0.45 | 0.56 |
|  | *p* | 0.09 | 0.22 | 0.41 | 0.09 | 0.02 | 0.004 |

Thereafter, the task-rest *t*-map of MCN was calculated, and PLS regression was adopted to map these MCN variations to anatomically patterned gene expression from the AHBA. We found that the PLS1 could explain 32% of the variance in the task-rest MCN differences (*p_perm_* = 0.01), close to that based on the D-K atlas. Then, we found that 837 PLS1+ (*Z* > 5) genes, and 1140 PLS1− (*Z* < −5) genes were significantly overexpressed in cortical regions, consisting of 1977 regional MCN gene list differences based on the Brainnetome atlas. To further validate the obtained gene list in our previous manuscript, a multigene-list meta-analysis [24] was conducted between the PLS1+ (PLS1-) gene lists obtained from the D-K and Brainnetome atlas. Notably, the gene lists derived from the two atlases were highly overlapped (left panel of Figure S11a and S11b). And more importantly, the enrichment pathways were also highly overlapped with only one pathway not matched (right panel of Figure S11a and S11b), further validating the generalized relationship between gene expression and the regional MCN alterations.


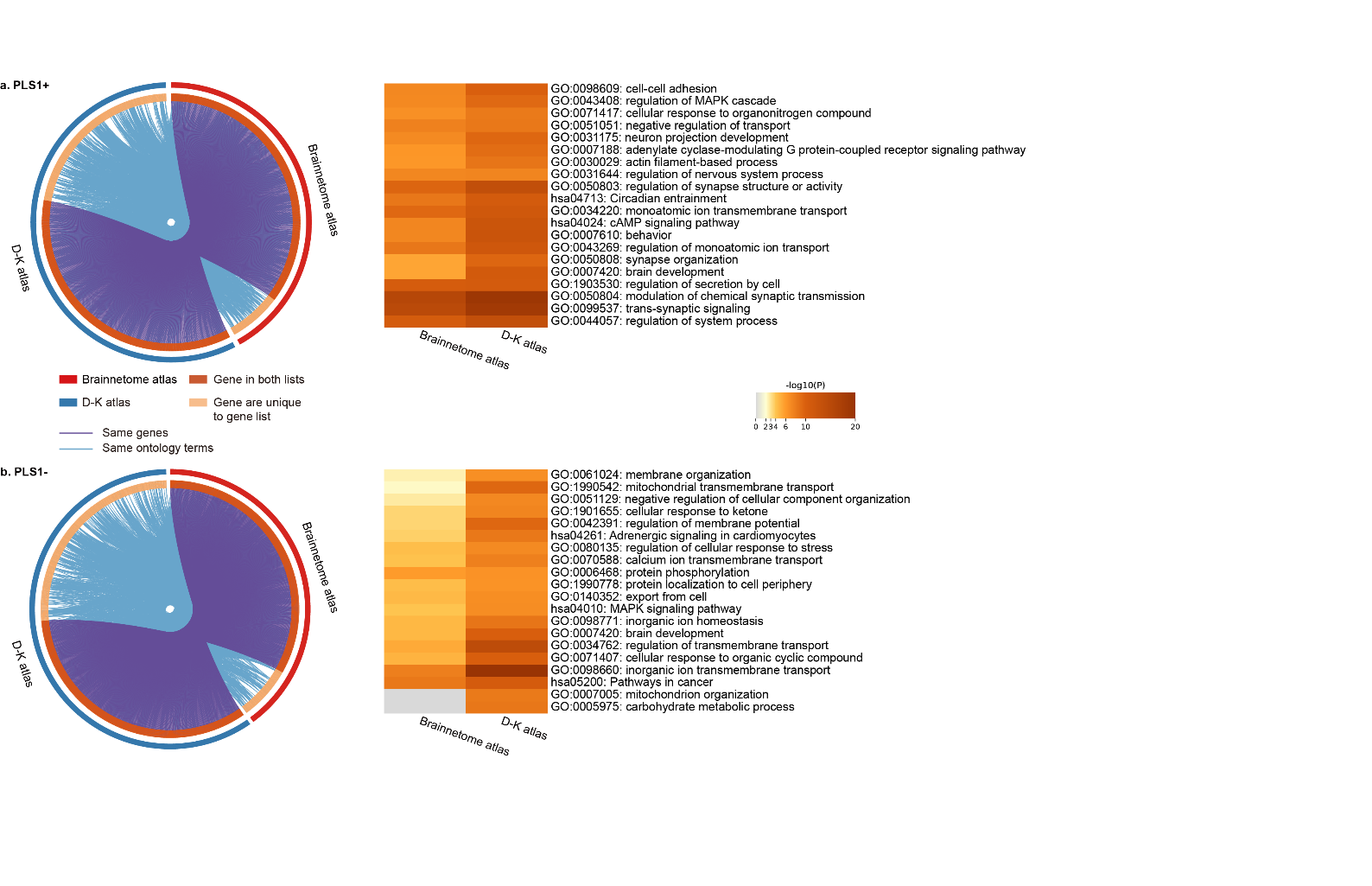


**Figure S11.** Validation of transcriptional enrichments of changes in MCN with **a.** PLS1 + and **b.** PLS1− weights, respectively. Circos plot of genes overlapped between the D-K and Brainnetome atlas. The right panel represents the overlapped ontology terms between the PLS1+ (PLS1-) genes derived from the D-K and Brainnetome atlas.
